# Increased Antidepressant Use after Intensive Care for Aneurysmal Subarachnoid Haemorrhage: A Machine-Learning-Enhanced Propensity-Matched Nationwide Cohort Study

**DOI:** 10.1007/s12028-026-02517-z

**Published:** 2026-05-04

**Authors:** Ryan Falck-Jones, Johan Olsson, Cecilia Åkerlund, Erik von Oelreich, Jesper Eriksson, Anders Oldner, Peter Alpkvist, Johan Lundberg, Mikael Svensson, Linn Hallqvist

**Affiliations:** 1 Department of Physiology and Pharmacology, Karolinska Institutet, Stockholm, Sweden; 2 Perioperative Medicine and Intensive Care, Karolinska University Hospital, Stockholm, Sweden; 3 Department of Clinical Neuroscience, Karolinska Institutet, Stockholm, Sweden; 4 Department of Neurosurgery, Karolinska University Hospital, Stockholm, Sweden; 5 Department of Neuroradiology, Karolinska University Hospital, Stockholm, Sweden; 6 Department of Paediatric Perioperative Medicine and Intensive Care, ECMO Centre Karolinska, Karolinska University Hospital, Stockholm, Sweden

**Keywords:** Subarachnoid hemorrhage, Mood disorders, Critical care

## Abstract

**Background:**

Mood disorders after aneurysmal subarachnoid haemorrhage (aSAH) are common. Meanwhile, mood disorders are also common after intensive care for any reason, and whether aSAH confers an excess risk remains unknown.

**Methods:**

In this retrospective study, patients with cases of aSAH admitted to intensive care in 2012–2023 were identified in nationwide registers and antidepressant (AD) prescriptions used as a proxy for mood disorders. The primary outcome was incidence of AD therapy in patients who were previously AD-naïve, along with attributable risk factors and impact on sick leave and disability pensions. To investigate the risk of AD treatment in aSAH exceeding what would be expected after intensive care, a control cohort of all patients in the intensive care unit (ICU) nationwide between 2012 and 2018 was identified. Using machine-learning-driven nearest-neighbour propensity score (PS) matching, the relative risk of AD treatment in ICU-treated aSAH cases vs. non-neurovascular ICU cases was calculated.

**Results:**

In all, 3149 patients with aSAH who were AD-naïve were identified, with a cumulative incidence of AD treatment within 1 year of 25.5% and markers of disease severity being the main risk factors for AD treatment. Patients with aSAH who were treated with ADs used significantly more sick leave and disability pensions than patients with aSAH who were not treated with ADs. After propensity score matching with a general ICU cohort, patients with aSAH still had a risk ratio (RR) of 1.333 [95% confidence interval (CI) 1.241–1.425] of AD treatment within 1 year, indicating an additional impact of aSAH on the development of mood disorders.

**Conclusions:**

Our results suggest patients with aSAH have a risk of mood disorders exceeding what would be expected after intensive care for non-neurovascular reasons.

**Supplementary Information:**

The online version contains supplementary material available at https://doi.org/10.1007/s12028-026-02517-z.

## Introduction

Aneurysmal subarachnoid haemorrhage (aSAH) is an uncommon subtype of haemorrhagic stroke with an estimated global incidence between 4 and 7 per 100,000 patient-years. It affects patients of all age groups, often with devastating consequences [1]. It is estimated that a quarter of patients die, and half of survivors are left with neurologic complications [2]. Frequently, aSAH causes significant cognitive impairment in survivors, and this is generally considered the primary sequelae [3]. Depression following aSAH also appears to be common, with a 2020 systematic review reporting a weighted frequency of depression following aSAH at 28% [4]. Meanwhile, depression is also common after intensive care treatment regardless of aetiology, with a 2024 study reporting a cumulative incidence of de novo antidepressant treatment 1 year after intensive care unit (ICU) admission of 15% [5–7]. This prompts the question of how much can be attributed to the pathophysiology of aSAH compared with the nature of intensive care treatment.

The association of various patient and disease characteristics and post-aSAH depression has been investigated in a wide range of studies with varying results. Occasionally, female sex has been associated with post-aSAH depression [8,9], along with younger age [10], ethnicity and non-fluency in the majority language [3]. Multiple studies have shown an association between prior psychiatric conditions and post-aSAH depression [3,8,11], along with a weak association with specific somatic comorbidities such as diabetes and chronic obstructive pulmonary disease [12]. Functional outcome measure tools such as the Modified Rankin Scale (mRS) and the Glasgow Outcome Scale (GOS) have consistently been shown to be correlated with depression [9,10,12], as has the occurrence of post-aSAH epilepsy [10]. Certain evidence also indicates an association between depression and aSAH-related infarcts and infarct or aneurysm location [3,13,14]. Moreover, at least two studies have reported a correlation between cortisol levels and depression [15,16]. Finally, indicators of more severe disease (e.g. increased Hunt–Hess score) have shown an association with post-aSAH depression [9,10].

The association between depression and more severe disease has also been evaluated in patients without aSAH who spent time in the ICU with varied results. In a recent study, poor physiology on ICU admission and longer ICU stay were clearly associated with depression 1 year after ICU admission, along with female sex and prior psychiatric conditions [6]. This finding is, however, not in line with results from two earlier systematic reviews, both finding no association between longer ICU stay or poorer physiology and depression [5,7].

Since depression appears to be common after intensive care for any reason, this raises the question of whether the observed increased incidence of depression after aSAH simply reflects the influence of disease severity or other factors related to intensive care, rather than an association with the disease itself. In the current study, we therefore investigate mood disorders after aSAH in a large nationwide cohort and specifically attempt to isolate and quantify any excess risk attributable to aSAH. By these means we aim to address the hypothesis of an inherent effect on the development of mood disorders resulting from aSAH.

## Methods

The study was performed as a register-based study including data for all adult patients in Sweden requiring neuro-intensive care between 2012 and 2023. The study was performed according to the Declaration of Helsinki and approved by the National Ethical Review Authority (Dnr: 2022-03054-01). The Strengthening the Reporting of Observational Studies in Epidemiology (STROBE) Reporting Guidelines were adhered to in the writing of the manuscript [17].

### Population Registers

The study was performed using pseudonymised population register data extracted from multiple registers and subsequently linked to provide patient-level data. Briefly, registers maintained by the Swedish Board of Health and Welfare included the National Patient Register (NPR), the Cause of Death Register (CDR) and the Prescribed Drug Register (PDR). Data from these registers were linked with data from the Swedish Intensive Care Register (SIR), the Longitudinal Integrated Database for Health Insurance and Labour Market Studies (LISA) and the Microdata for Analysis of Social Insurance (MiDAS) register maintained by the Swedish Social Insurance Agency. A detailed description of the population registers used in the study is provided in Supplementary Materials, section 1.

### Study Cohort Selection Strategy

**Fig. 1:**
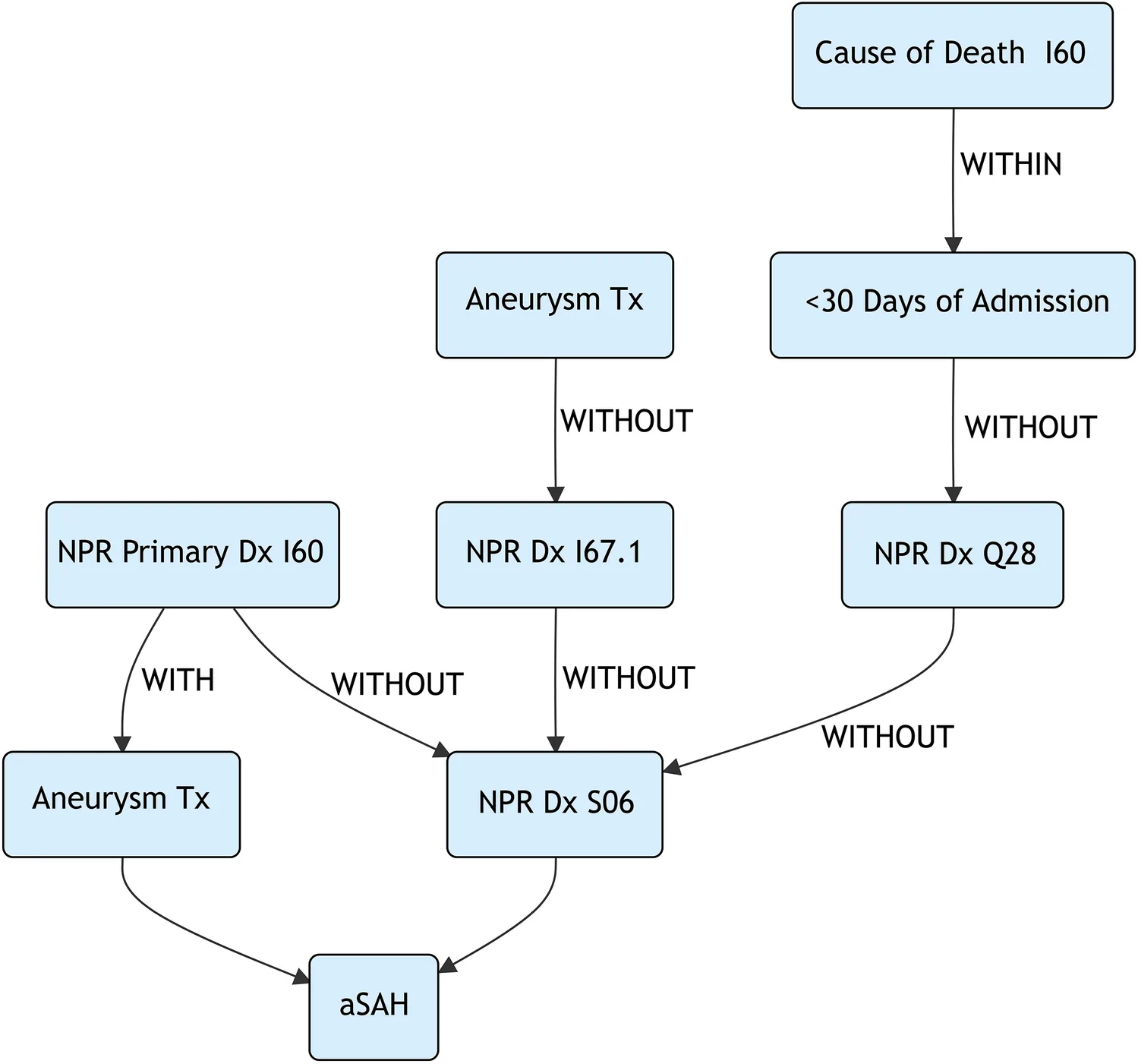
Identification of aSAH in register data. ICD-10 codes: I60 (Subarachnoid haemorrhage), I67.1 (Non-ruptured cerebral aneurysm), Q28 (Other congenital malformations of circulatory organs), S06 (Intracranial injury). aSAH, aneurysmal subarachnoid haemorrhage; NPR, National Patient Register; Aneurysm Tx, clipping/coiling of aneurysm

Using the nationwide study cohort, aSAH cases were subsequently selected according to the steps shown in Fig. 1 [detailed structured query language (SQL) source code available Supplementary Materials, section 3]. Case ascertainment was based on a combination of an International Classification of Diseases Version 10 (ICD-10) diagnosis I60 [i.e. subarachnoid haemorrhage (SAH)] in the NPR, aneurysm treatment identified by coding of interventions and cause of death coding in the CDR (Fig. 1).

Following identification of aSAH, patients with a dispensed antidepressant prescription within 180 days before ICU admission were excluded, creating an antidepressant-naïve aSAH cohort. Within this cohort, patients surviving less than 365 days after ICU admission were censored to mitigate the competing risk of death when investigating post-ICU antidepressant prescription.

### Data Management and Statistical Analysis

Raw register data were pre-processed to create continuous ICU admissions, regardless of administrative admission coding or transfers between ICUs. Stacked ICU admissions were considered a single continuous admission if the period between ICU discharge and re-admission was less than 12 h. Patient-level comorbidity information up to 5 years before ICU admission was compiled using the NPR, with diagnoses such as diabetes being expanded using evidence of treatment extracted from the PDR (Supplementary Materials, section 2). The Charlson Comorbidity Index (CCI) score was calculated for each patient using the method described by Ludvigsson and colleagues [18].

### Descriptive Statistics

Descriptive statistics for both the study cohort and the control cohort were calculated using appropriate statistical measures and presented in table format. The choice of summary statistic and comparative test was determined after assessing the distributions of continuous variables. For continuous parameters with approximately normal distribution, parametric statistics and testing were used. Age was found to have a multimodal distribution in the control cohort and was therefore presented as a median, with comparison between groups analysed using an asymptotic two-sample Kolmogorov–Smirnov test in this table, whereas it was presented as a mean with standard deviation for the study cohort. Both ICU length of stay (ICU LOS) and CCI score were found to be highly skewed and therefore transformed prior to statistical testing. ICU LOS was binned into four approximately equally sized bins (bounded by the closest integer) and imputed as a nominal variable, while CCI scores were binned into three bins with CCI scores of 0, 1 and > 1, respectively. For further details on the distribution of baseline characteristics, see Supplementary Materials, Fig. S4.1.

Additionally, a point-prevalence graph was created by querying the PDR for dispensed antidepressants on each day for a period of 2 years before and 2 years after ICU admission. If a prescription was dispensed within 90 days prior to the query date, the patient was considered to have ongoing antidepressant treatment; if > 90 days had elapsed without a dispensed prescription, the patient was no longer considered to have ongoing treatment. The choice of 90 days is in accordance with Swedish prescription regulations, which allow for iterated prescriptions to be dispensed for a maximum period of 90 days.

### Missing Data

Examination of the data for the study cohort revealed little missing data, except for education level, which was not extracted for 2023–2024 and was therefore considered to be missing at random (MAR) only with respect to calendar year and missing completely at random (MCAR) with respect to all other variables. Data for certain ICU variables were sporadically not reported, with variation across centres. The degree of missingness in this data was low but clearly related to other predictors and therefore not MCAR. A more detailed description of missing data is available in the Supplementary Materials, section 5. In general, missing data were imputed using multiple imputation using chained equations (MICE), estimated using the random Forest (RF) method for five imputations over five iterations and pooled according to Rubin’s rules [19].

Missing data from the much larger control cohort were instead handled using listwise deletion (i.e. complete case analysis). In analyses using combined data from both cohorts the complete cases from the control cohort were joined with each of the five imputed datasets of for the study cohort, yielding five combined datasets.

### Logistic Regression and Variable Selection

Examination of covariates influencing the odds of antidepressant therapy within a year of ICU admission was performed in the study cohort using a multivariable logistic regression model, yielding adjusted odds ratios. Covariate selection was guided by domain knowledge, and initial covariate screening was performed using a univariate logistic regression (Supplementary Materials, Table S6.2). Collinearity between potential covariates was assessed by calculating Pearson’s ρ for continuous variables, Cramér’s V for nominal variables and a point-biserial correlation coefficient for a nominal-continuous interaction (Supplementary Materials, Fig. S6.1, Table S6.1). Additionally, domain knowledge was used to logically exclude variables containing the same data component. Specifically, the Simplified Acute Physiology Score 3 (SAPS 3) was calculated using age and consciousness level and was therefore manually recalculated after dropping these variables from the calculation (Supplementary Materials, section 6). Finally, absence of gross multicollinearity was confirmed by the generalised variance inflation factor remaining under 2 for all variables [20]. Additionally, ordinal variables were recoded into nominal values to enable interpretation of odds ratios relative to a reference level rather than modelling ordinal data as orthogonal polynomial splines. Diagnostic details of the multivariate logistic regression were subsequently analysed to evaluate the fulfilment of modelling assumptions (Supplementary Materials, Figs. S6.2, S6.3). Finally, a 2000-times bootstrap resample was drawn from the original incomplete dataset, and within each resample missing data were imputed five times using MICE [21]. The logistic regression model was fitted to each imputed dataset and pooled using Rubin’s rules [19], yielding one estimate per bootstrap. The resulting adjusted odds ratios along with the 95% confidence intervals (CIs) were then calculated across the bootstrap distribution of pooled estimates using the percentile method.

### Analysis of Implications for Workforce Participation

Evaluation of the implications of post-ICU AD treatment on workforce participation was assessed by investigating the difference in sickness absence (SA) and disability pension (DP) in the same individual within a 1-year window preceding ICU admission and within a 1-year window between years 1 and 2 after ICU admission. Patients were censored if they were unable to have a full observation period due to mortality < 2 years after ICU admission or where ICU admission occurred less than 2 years before the end of the study. Furthermore, this analysis was restricted to patients 60 years or younger at the time of ICU admission. Using data from the Swedish Social Insurance Agency, all periods of SA or DP were collected. For each day during the observation period, a leave factor was calculated by adding any SA or DP occurring on that day. In the case of parallel SA and DP periods with a total sum of leave exceeding 100%, the leave factor was set to 100%. The sum of leave factors across the two observation periods therefore constituted the net total of annual leave, and the difference between the two windows was calculated for each patient, referred to as the Δleave. The distribution of Δleave was multimodal and therefore was plotted as a cumulative distribution in the groups with or without AD treatment. Finally, the two distributions were compared using an asymptotic two-sample Kolmogorov–Smirnov test with *p*-values based on a 10,000-times Monte–Carlo simulation.

### Matching Process

To estimate the marginal effect of aSAH on the risk of antidepressant therapy within a year after ICU admission, the study population was propensity score matched with a general ICU population. The control population used for matching comprised all patients admitted to an ICU nationwide during 2012–2018. Data availability for the control group was almost identical to that of the study population, except for data on surgical procedures such as aneurysm clipping and endovascular aneurysm treatment. Due to the lack of these data, the case ascertainment strategy for identifying patients treated for aSAH in the control population had to be less precise than in the main study cohort. The final control population therefore was attained by removing all admissions with a neurovascular diagnosis as the primary cause of admission. Following this exclusion, data were processed in the same way as for the main study cohort, with administrative admissions aggregated into unique continuous ICU admissions.

Propensity scores denoting the probability of cohort membership (study or control cohorts) were subsequently calculated for each admission in the combined data set using a random Forest classification model on each of the five imputations performed on the study-population data joined with the control cohort following listwise deletion. First, 20% of the data was reserved for a holdout set, while the model was trained on the remaining 80%. Hyperparameter tuning across a five-fold cross-validation resample of the training subset was performed by using a space-filling grid search with the Latin Hypercube method and a grid size of 20. The hyperparameter settings maximising the area under the receiver operating characteristic curve (AUROC) were selected, and the final model was trained. Investigation of ROC curves for each cross-validation fold revealed minimal overfitting. Subsequent model testing on the 20% holdout set was performed, analysing accuracy, Brier score and AUROC, along with plotting the ROC curve for each imputation (Supplementary Materials, section 8). Finally, the propensity scores were predicted for the entire data set.

Using the propensity scores generated using the random Forest model described above, 3:1 nearest-neighbour matching without replacement on the logit of the propensity score was performed, along with exact matching on female sex. Various matching specifications were assessed to provide an optimal balance of covariates, based on minimising the absolute standardised mean deviation (ASMD) and the Kolmogorov–Smirnov statistic for each covariate and on minimising the average ASMD across all covariates. Additionally, balance diagnostics such as the estimated cumulative distribution function (eCDF) and covariate distributions were assessed both visually and numerically to identify the optimal balance of covariates, along with the ASMDs for interactions between covariates and their squares. Detailed balance diagnostics are available in the Supplementary Materials, section 9.

The 3:1-matched dataset was subsequently used to estimate the marginal effect of aSAH on the risk of antidepressant therapy within 1 year of ICU admission by fitting a multivariable logistic regression model and pooling across imputations, yielding a doubly robust estimate of the average treatment effect in the treated (ATT). Finally, bootstrapped confidence intervals were calculated from a 2000-times bootstrap resample for each imputation, and the resulting pooled metrics were presented as risk ratios (RRs) along with their bootstrap distributions.

### Sensitivity Analyses

Sensitivity analyses are reported in Supplementary Materials, section 7. Briefly, analysis investigating the effect of various cut-offs in the definition of patients who were antidepressant-naïve showed no influence on the incidence of post-ICU AD prescription (Supplementary Fig. S7.1). Additionally, analyses of negative pharmacological controls (statins and thyroxin) showed a retained linear trend in these prescriptions over time (Supplementary Fig. S7.2).

#### Software Environments

All statistical analysis and modelling were performed using R 4.5.1 [22] and Positron [23]. Additionally, SQLite [24] was used for data cleaning prior to analysis. For individual packages used, see Supplementary Materials, section 10. The source code is freely available via a public repository.

## Results

### Study Population

**Fig. 2:**
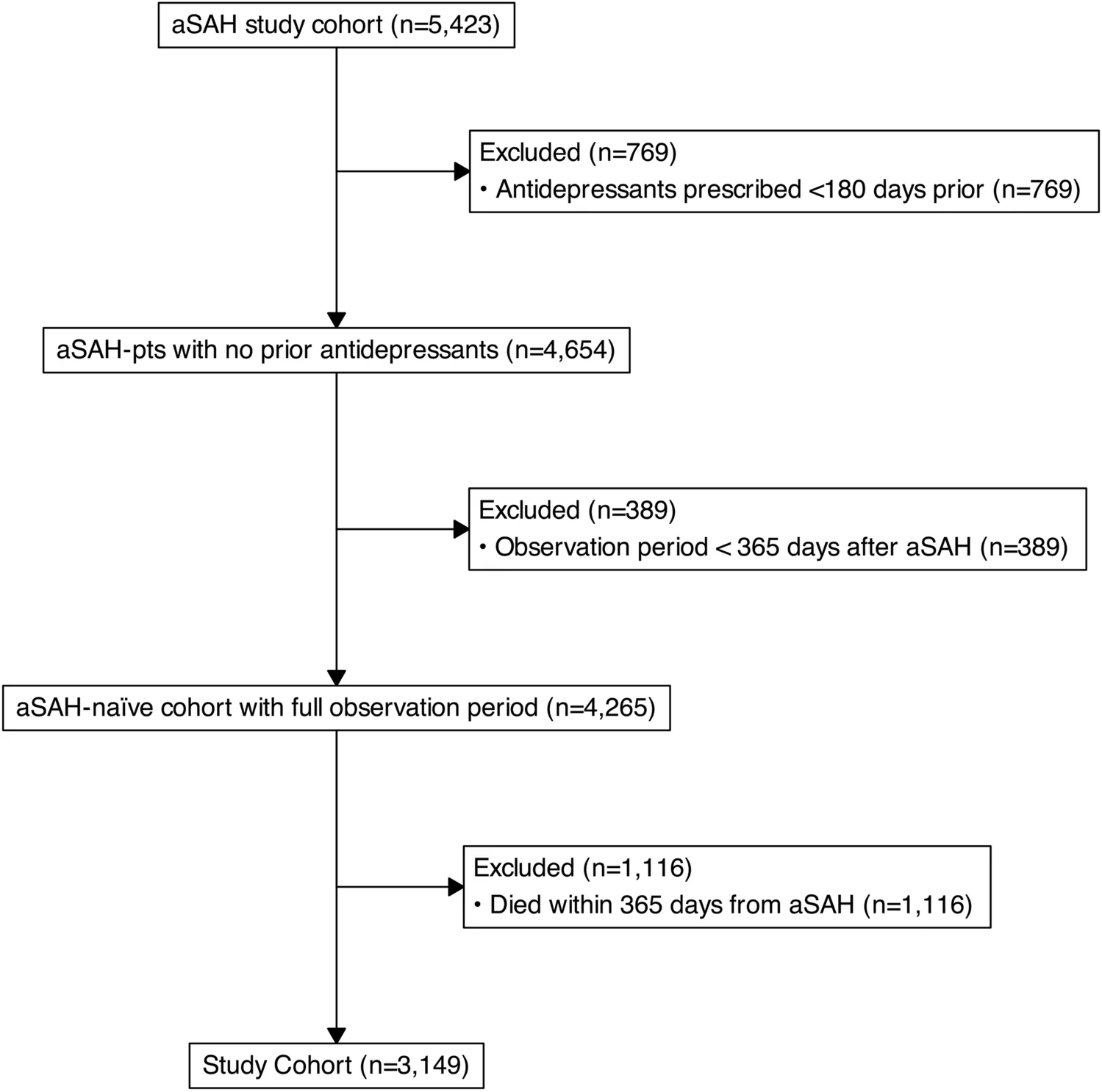
Flow diagram of study cohort

In total, 58,550 unique patients were extracted from the NPR (Fig. 2). Following the selection strategy described in the "Methods" section, a study cohort of 5423 patients with a presumed aSAH was identified. Of these patients, 769 had been dispensed a prescription of antidepressants in the 180 days prior to ICU admission for aSAH and were thus excluded from final analysis, along with 389 patients whose ICU admission occurred less than 1 year prior to the end of the study and 1116 patients who died within 1 year following ICU admission. The remaining cohort consisted of 3149 patients with aSAH surviving for 1 year after aSAH and without prior antidepressant therapy.

### Prevalence of Antidepressant Therapy

**Fig. 3:**
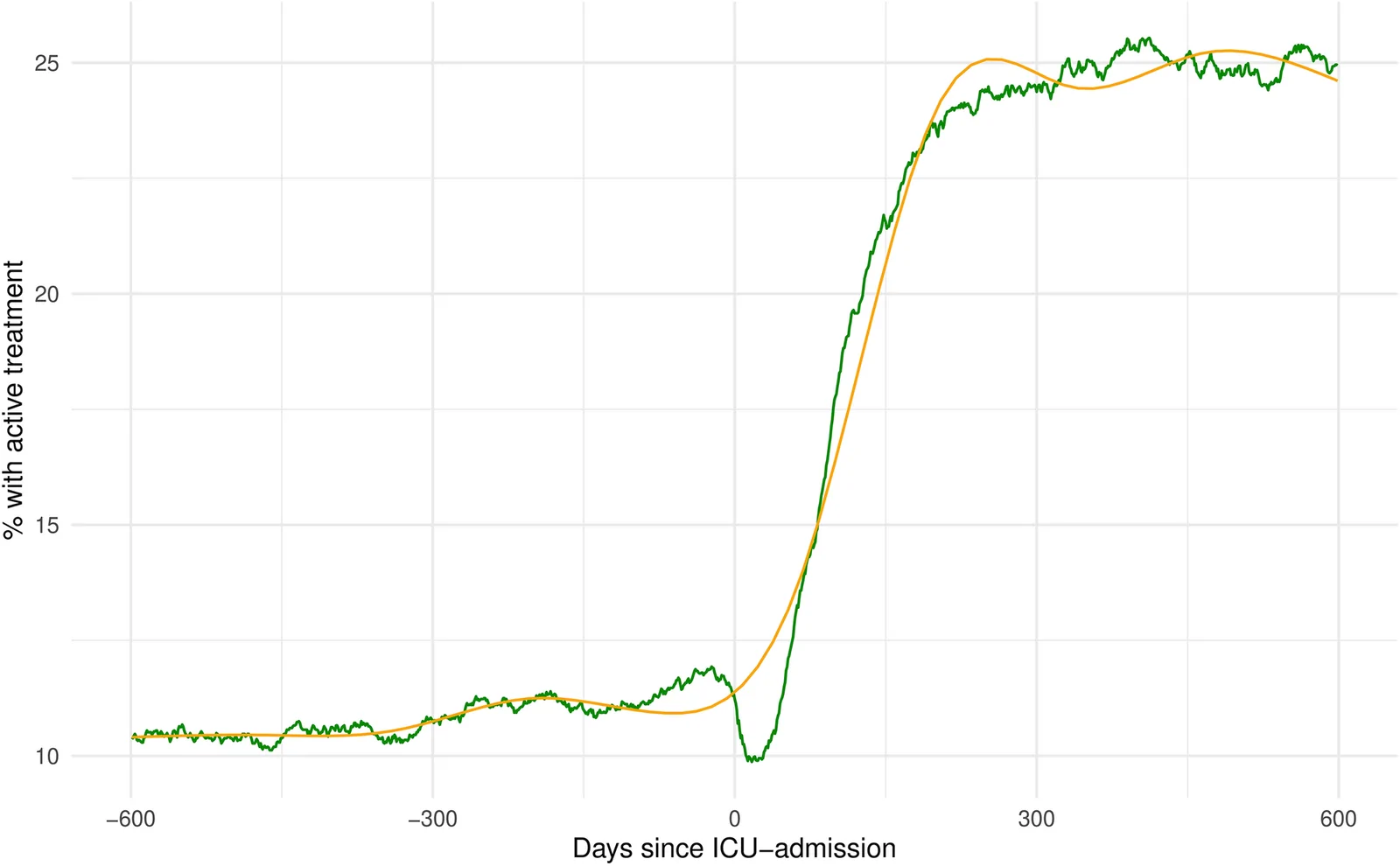
Point prevalence of antidepressant treatment. Point prevalence of ongoing antidepressant therapy plotted over time relative to ICU admission. Smoothing with a generalised additive model (yellow)

The point prevalence of active antidepressant treatment was assessed for all 5423 patients treated for aSAH by investigating whether an antidepressant prescription had been issued within the 3 months preceding the index date. Plotting point prevalence versus time relative to ICU admission demonstrated a sustained increase in the prevalence of antidepressant therapy after aSAH (Fig. 3).

### Baseline Characteristics of the AD-Free Cohort

Baseline characteristics of patients included in the analysis are presented in Table 1. As expected, there was a female predominance in both groups, but this was significantly more pronounced in patients treated with antidepressants after aSAH than in individuals who were not treated. There was a clear difference in ICU-related characteristics between groups, with a higher proportion of immediate invasive ventilation and poorer initial consciousness level in patients who subsequently received antidepressants.

**Table 1:** Baseline characteristics of study cohort (i.e. antidepressant-naïve aSAH cohort) stratified by existence of an antidepressant prescription within 1 year after ICU admission

Post-ICU AD-treatment	No	Yes	*p*-value^a^
	*N* = 2547	*N* = 872	
*Demographics*			
Female	1442 (57%)	544 (62%)	0.003
Age, mean (SD)	58 (13)	57 (13)	0.3
Education level			0.12
Primary and lower secondary	466 (21%)	158 (21%)	
Upper secondary	1032 (47%)	392 (51%)	
Tertiary	681 (31%)	211 (28%)	
Research degree	25 (1.1%)	5 (0.7%)	
*Comorbidities*			
Hypertension	557 (22%)	201 (23%)	0.5
Diabetes	149 (5.9%)	56 (6.4%)	0.5
Mood disorder	15 (0.6%)	11 (1.3%)	0.048
Anxiety disorder	13 (0.5%)	3 (0.3%)	0.8
Psychotic disorder	3 (0.1%)	2 (0.2%)	0.6
Substance abuse	37 (1.5%)	21 (2.4%)	0.059
CCI score			< 0.001
0	2115 (83%)	671 (77%)	
1	277 (11%)	145 (17%)	
> 1	155 (6.1%)	56 (6.4%)	
*ICU characteristics*			
Initial consciousness level			< 0.001
I (GCS ≥ 13)	1828 (75%)	466 (56%)	
II (GCS 7–12)	368 (15%)	214 (26%)	
III (GCS 6)	68 (2.8%)	44 (5.3%)	
IV (GCS 5)	43 (1.8%)	31 (3.7%)	
V (GCS ≤ 4)	131 (5.4%)	81 (9.7%)	
SAPS 3, mean (SD)	49 (11)	53 (12)	< 0.001
ICU LOS (days), median (Q1, Q3)	4 (1, 10)	7 (1, 17)	< 0.001
Invasive ventilation during ICU	2105 (83%)	771 (88%)	< 0.001

Data presented as *n* (%) unless otherwise specified. CCI, Charlson Comorbidity Index; GCS, Glasgow Coma Scale; ICU, intensive care unit; LOS, length of stay; Q1, first quartile; Q3, third quartile; SAPS 3, Simplified Acute Physiology Score 3; SD, standard deviation

^a^Pearson’s chi-squared test; Welch two-sample *t*-test; Fisher’s exact test; Wilcoxon rank-sum test

### Risk Factors for Post-aSAH Antidepressant Therapy

**Fig. 4:**
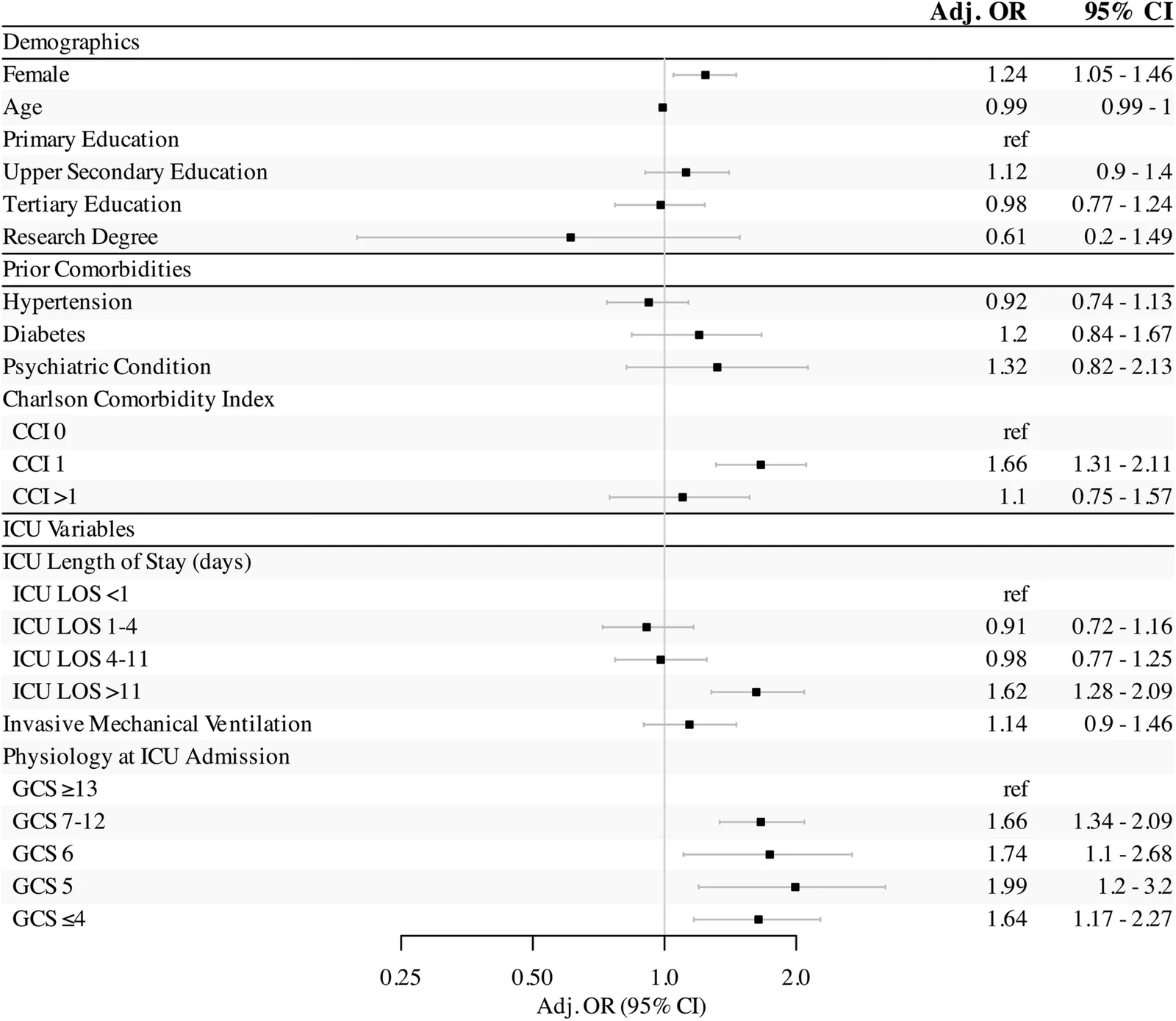
Multivariable logistic regression model. Forest plot of adjusted odds ratios calculated using a logistic regression model fitted to a 2000-times bootstrap resample with 95% confidence intervals. Model further adjusted for recalculated SAPS 3 after removal of age and consciousness level ("Methods" section), along with calendar year. “Psychiatric Condition” includes mood, anxiety, psychotic and substance abuse disorders

To investigate potential risk factors for post-ICU antidepressant therapy in patients with aSAH, a multivariable logistic regression model was created (Fig. 4). The model was adjusted for calendar year to account for temporal influences on antidepressant prescribing, along with a recalculated SAPS 3 after removing age and consciousness level. Female sex was found to be a statistically independent predictor of post-ICU AD treatment. No comorbidities were identified as independent predictors, and the CCI did not influence the odds of AD therapy. The main risk factors identified in the model were markers of disease severity, with decreasing consciousness level on admission along with increasing ICU length of stay predicting higher odds of AD therapy after aSAH.

### Analysis of Implications for Workforce Participation

**Fig. 5:**
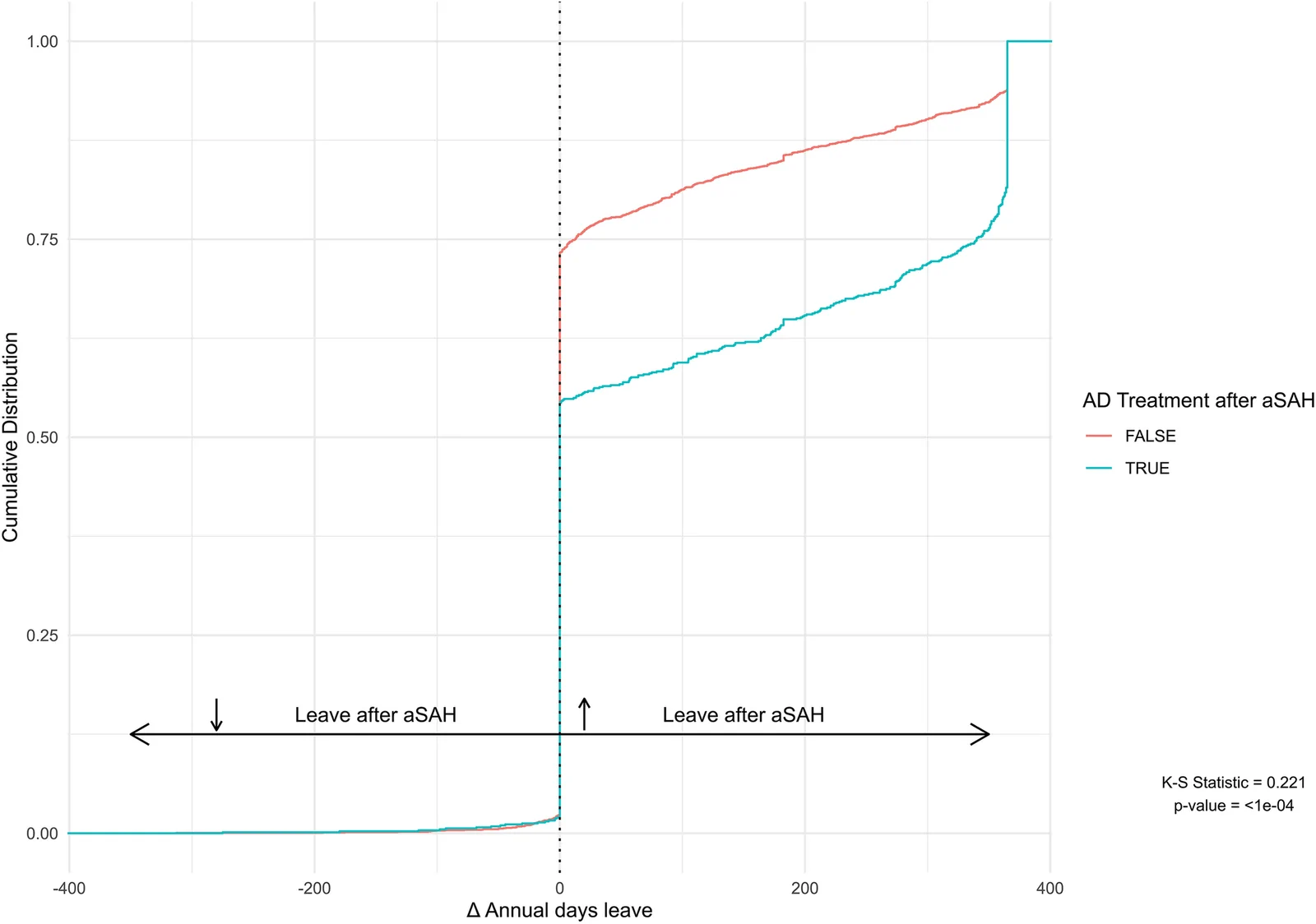
Cumulative distribution of the individual change in annual sick leave between days − 365 and − 1 relative to ICU admission and days + 366 and + 730 relative ICU admission (each a 1-year observation window). Statistical comparison of distributions using the Kolmogorov–Smirnov test showing significantly different distributions. *Reading guide:* Just over 1% of patients have a negative Δleave (i.e. more absence before aSAH than after)

Using data from the Swedish National Insurance Agency, the impact of mood disorders following aSAH on workforce participation was assessed. Patients requiring AD after aSAH had an apparent increase in the amount of sickness absence between 1 and 2 years post-aSAH when each individual was compared with themselves. This difference was statistically significant, and the cumulative distribution is plotted in Fig. 5.

### Comparison with the General ICU Population

To evaluate any additional risk of post-ICU AD treatment in patients with aSAH, the study cohort was matched to a cohort consisting of all patients in the ICU with non-neurovascular primary causes of admission in Sweden between 2012 and 2018. A comparison of baseline demographics, comorbidities and ICU variables is shown in Table 2. Of note, patients in the control cohort were predominantly male and were both older and more comorbid than patients in the study cohort. Additionally, ICU length of stay was shorter in controls, and there were less severe markers of disease severity.

**Table 2:** Patient characteristics in the aSAH cohort compared with the control cohort of patients in the ICU for non-neurovascular reasons 2012–2018

Characteristic	Study cohort	Control cohort	*p*-Value^a^
	*N* = 3419	*N* = 138,911	
*Demographics*			
Female	1986 (58%)	51,451 (37%)	< 0.001
Age, median (Q1, Q3)	58 (50, 67)	64 (46, 73)	< 0.001
Education level			< 0.001
Primary and lower secondary	624 (21%)	39,288 (34%)	
Upper secondary	1424 (48%)	51,979 (45%)	
Tertiary	892 (30%)	23,241 (20%)	
Research degree	30 (1.0%)	763 (0.7%)	
*Comorbidities*			
Hypertension	758 (22%)	37,832 (27%)	< 0.001
Diabetes	205 (6.0%)	18,474 (13%)	< 0.001
Psychiatric condition	92 (2.7%)	20,430 (15%)	< 0.001
*CCI*			< 0.001
0	2786 (81%)	82,587 (59%)	
1	422 (12%)	23,284 (17%)	
> 1	211 (6.2%)	33,040 (24%)	
ICU characteristics			
SAPS 3, mean (SD)	50 (11)	50 (14)	< 0.001
*Initial consciousness level*			< 0.001
I (GCS ≥ 13)	2294 (70%)	89,704 (78%)	
II (GCS 7–12)	582 (18%)	16,149 (14%)	
III (GCS 6)	112 (3.4%)	2736 (2.4%)	
IV (GCS 5)	74 (2.3%)	1080 (0.9%)	
V (GCS ≤ 4)	212 (6.5%)	5403 (4.7%)	
*ICU LOS* (*days*)			< 0.001
0–1 days	870 (25%)	70,595 (51%)	
1–4 days	766 (22%)	48,251 (35%)	
4–11 days	920 (27%)	13,659 (9.8%)	
> 11 days	863 (25%)	6168 (4.4%)	
*Outcome*			
Antidepressant treatment	872 (26%)	18,423 (13%)	< 0.001

Data presented as *n* (%) unless otherwise specified. CCI, Charlson Comorbidity Index; GCS, Glasgow Coma Scale; ICU, intensive care unit; LOS, length of stay; Q1, first quartile; Q3, third quartile; SAPS 3, Simplified Acute Physiology Score 3; SD, standard deviation

^a^Pearson’s chi-squared test; asymptotic two-sample Kolmogorov–Smirnov test; Welch two-sample *t*-test

**Fig. 6:**
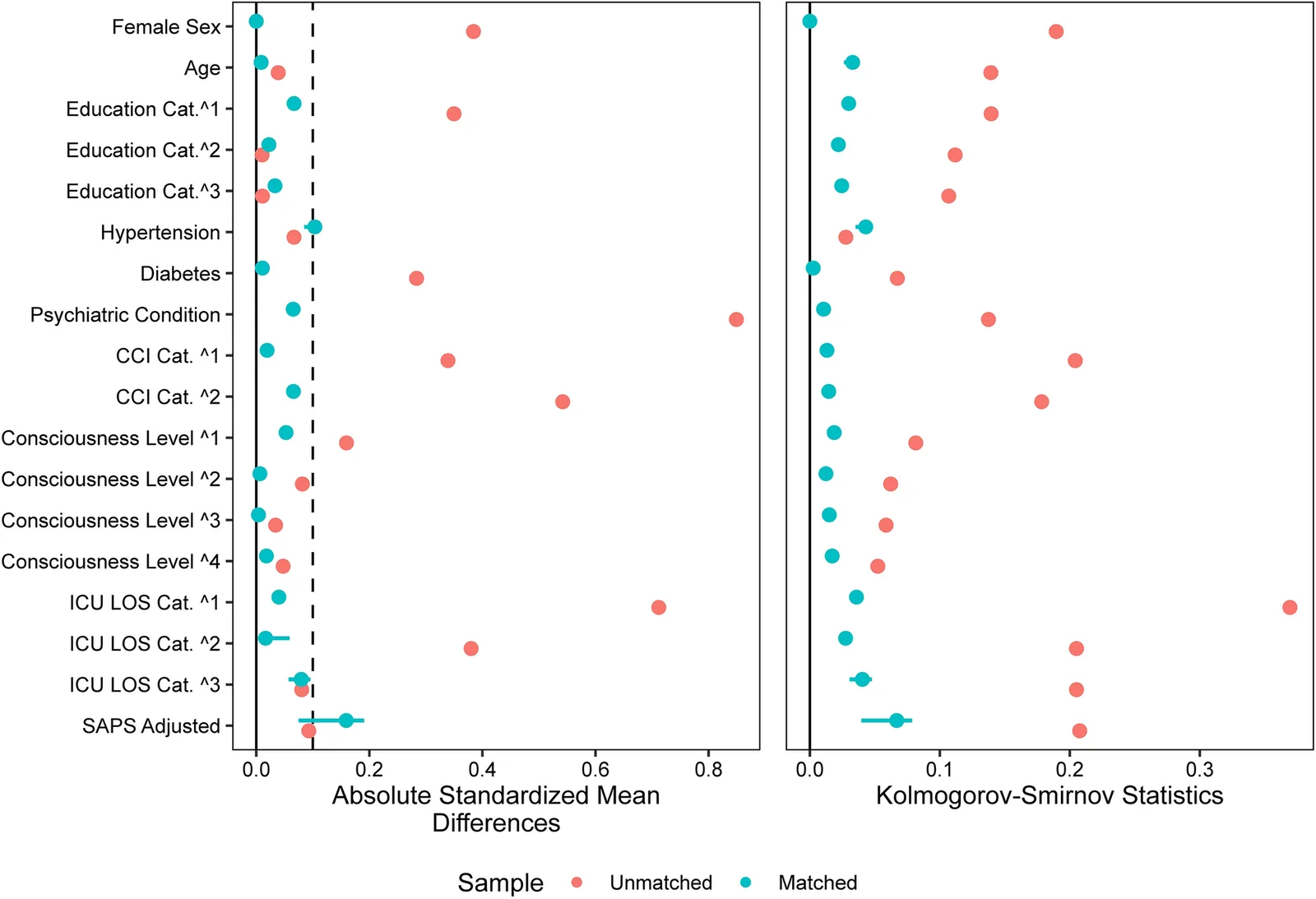
Balance plot before and after matching across imputations. Ordinal variables modelled as orthogonal polynomial splines to retain non-linear ordinal effects. See Table 2 for the underlying categories

Considering the significant differences between the study and control cohorts, especially with regard to markers of disease severity, a propensity score matching process was undertaken to evaluate the marginal effect of aSAH on the risk of post-ICU AD treatment relative to the risk conferred by exposure to intensive care alone. The matched dataset demonstrated substantial improvement in covariate balance (Fig. 6), with a mean absolute standardised mean difference after matching below 0.05 (Supplementary Fig. S9.3), indicating excellent covariate balance [25]. Finally, a logistic regression model was fitted to the matched dataset.

**Fig. 7:**
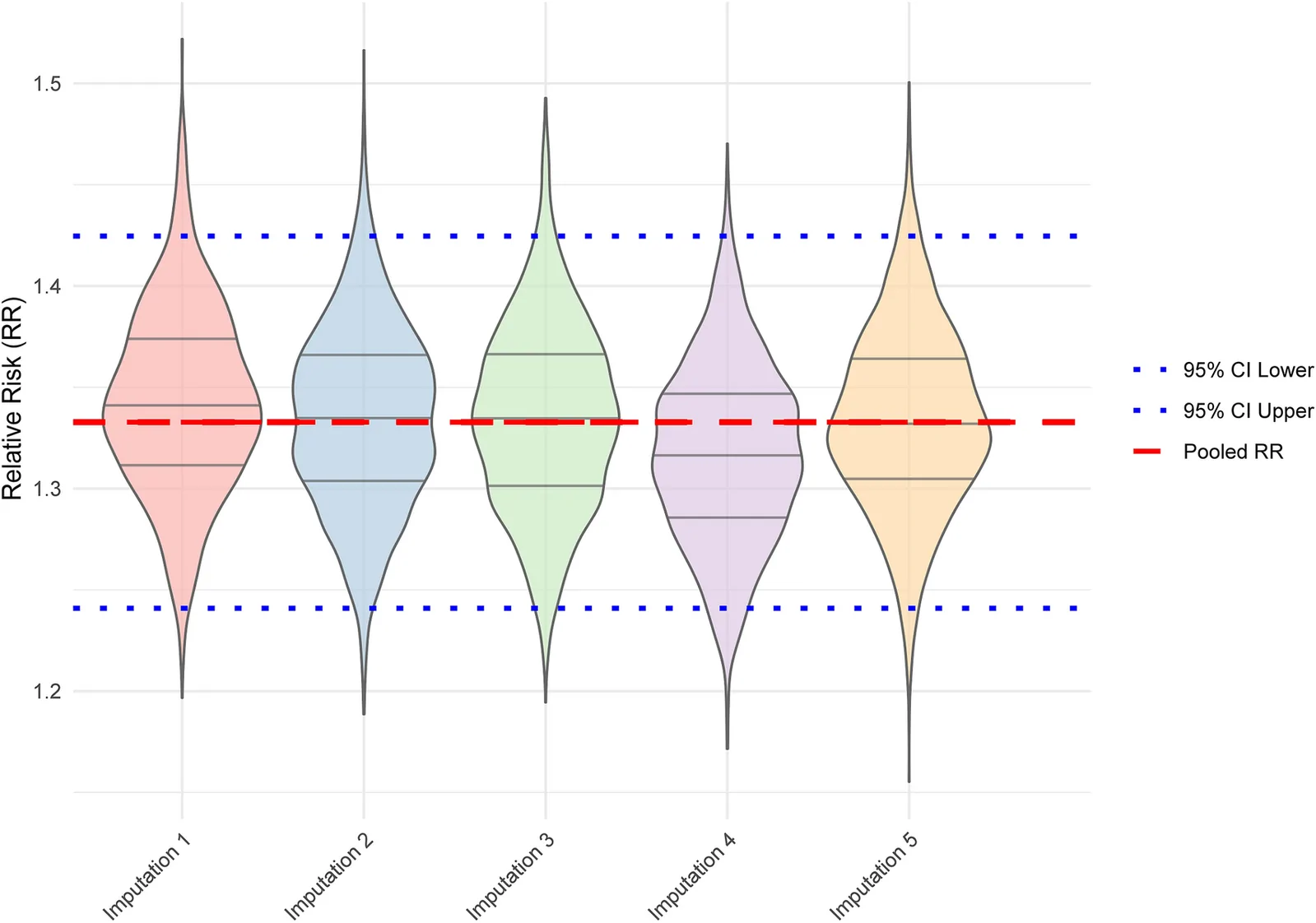
Relative risk of post-ICU AD treatment across imputations. Violin plots of 2000-times bootstrap resample density distribution of attributable relative risk of post-ICU AD treatment in patients with aSAH compared with matched patients in the ICU for non-neurovascular reasons for each imputation. Pooled relative risk and 95% CIs across imputations

The model fitted to the matched dataset revealed a significantly increased, doubly robust estimate of the marginal effect of aSAH + intensive care versus intensive care alone (RR 1.33; 95% CI 1.24–1.43) (Fig. 7).

## Discussion

In this study, we have investigated mood disorders after intensive care for aSAH by assessing antidepressant prescriptions in a nationwide dataset with almost complete coverage. We show an increased incidence of AD therapy after aSAH, with markers of disease severity identified as the main predictors, suggesting the risk of mood disorders may simply be a result of more severe disease. However, using machine-learning-driven propensity score matching, we have demonstrated that there is, in fact, an increased risk of post-ICU AD therapy in aSAH compared with other non-neurovascular aetiologies. This result supports an inherent, albeit pathophysiologically unknown, effect of aSAH on the development of mood disorders extending beyond simply being a proxy of disease severity.

Depression and other mood disorders after aSAH have previously been studied using various methodologies. Defining the outcome of depression frequently rests on the result of interview-based methods, the most common being the Hospital Anxiety and Depression Scale (HADS) [4]. An alternative approach is to evaluate trends in the prescription of antidepressant therapy, demonstrated in a 2016 case–control study [10]. In studies evaluating mood disorders after general ICU care, diagnoses documented as part of clinical routine are also used [26]. Likewise, the case ascertainment strategy for identifying aSAH is handled differently depending on study design. In prospective studies, identification is typically performed by clinical diagnosis using imaging and/or lumbar puncture [3]. Retrospective research, however, poses challenges in identification of SAH and isolation of *aneurysmal* SAH, and in many studies, data from prospectively registered trials are used [12,27]. Alternatively, population registers can be used to identify cases, but this is dependent on register quality [28].

Importantly, our method only allows for investigation of AD treatment and not the actual occurrence of mood disorders, clearly introducing a risk of bias. Using treatment as a proxy of disease is methodologically simple and allows for the use of large data in an efficient way when compared with using diagnostic and interview-based methods to ascertain the presence of mood disorders. Notwithstanding, potential differences in post-ICU follow-up between the study and control cohorts pose a significant threat of introducing bias, as patients with aSAH are generally meticulously followed after their initial insult and could have a lower threshold for the diagnosis and treatment of mood disorders when compared with patients in the ICU in general. For this reason, we conducted a sensitivity analysis using pharmacologic controls assumed to be unrelated to aSAH (statins and thyroxin). This analysis supported a null hypothesis of a retained unaffected linear trend in these prescriptions over time in contrast to the exponential increase seen in AD prescription following aSAH. This result therefore argues against improved follow-up as the sole driver of increased AD prescription after aSAH and supports our conclusion.

Despite these important concerns, we conclude that patients with aSAH have an increased risk of post-ICU AD treatment and likely also have an increased incidence of depression compared with patients in the ICU in general. These findings are also consistent with prior research from both Finland [10] and Sweden [9], demonstrating an increased use of AD after aSAH in the former study and a reduced quality of life and more anxiety/depression in the latter. However, in contrast to these studies in which aSAH was compared with a population reference group, we describe an increased AD use in excess of what would be expected simply as a result of critical illness and intensive care. Since previous research has shown an increase in AD treatment after intensive care for any reason [5,6], one would expect this increase in AD treatment to also occur in the setting of aSAH. Our findings are therefore indicative of some additional pathophysiologic effect associated specifically with neurovascular diseases and potentially aSAH.

In addition to evaluating the presence of mood disorders after aSAH, we also aim to place this finding in a patient-centred context by investigating the effects of post-ICU mood disorders on sickness absence (SA) and disability pensions (DP). In this respect, we are able to both replicate the previous studies demonstrating a stark increase in SA and DP after aSAH and show that this increase is more pronounced in patients treated with AD after aSAH. Since we have elected to view SA and DP as outcomes resulting from mood disorders, we have chosen to evaluate a time frame beginning 1 year after the onset of disease. While this approach allows for a correct temporal analysis, we were still unable to assess causality, and our results should therefore be interpreted with caution. Regardless, our findings do highlight the association between mood disorders and socio-economic consequences with profound patient-centred implications.

### Choice of Statistical Analysis

Aside from the clinical relevance of investigating mood disorders after aSAH, the current study also serves as a real-life example of using machine learning methods to improve propensity score matching and enable quasi-causal inference in non-randomised retrospective research. Since the estimation of propensity scores constitutes a classification problem, and the specific coefficients are unimportant for the analysis, we opted for a meta-classification and regression tree (CART)-based method known as random Forests, which itself is an ensemble method consisting of multiple decision trees trained on slightly different data which are combined to increase performance and mitigate problems of overfitting. The use of a classification model rather than a logistic regression model removes several limitations associated with parametric models, such as the requirement of a linear relationship between the independent variables and the log-odds of the dependent variable. Previous research has shown that logistic regression is often used to estimate propensity scores, with little consideration given to evaluating interactions and assumptions necessary for correct model fitting [29]. While logistic-regression-based propensity scores generally perform adequately, machine learning algorithms and specifically ensemble CART methods, offer advantages by providing greater flexibility, improving covariate balance and reducing bias [30, 31]. In our analyses, we also show high model performance in predicting cohort membership with very high accuracy and AUROC along with a low Brier score. This high performance was also retained across all cross-validation folds, indicating little overfitting. Using the resulting PS from the RF model as a measure of distance, 3:1 nearest-neighbour matching was performed, a logistic regression was fitted to the matched data and the ATT was calculated. In this way, we demonstrate an additional marginal effect of aSAH on the risk of subsequent antidepressant therapy, which goes beyond the risk incurred by intensive care alone.

### Strengths and Limitations

Aside from ascertainment of cases and outcomes discussed above, the study design also introduces several inherent limitations which should be noted when interpreting our results. Firstly, this study was performed in a single high-income country with a relatively homogenous population, thus reducing the external validity and generalisability of results to settings with different demographic distribution. While this is an important aspect to consider, Sweden is also unique in having high-quality population registers linked on an individual level via the personal-identity-number system, enabling individual-level data for the entire population of approximately 10 million and thus providing a source population of considerable size. Secondly, data availability in our population registers restricted the selection of variables able to be included in the study, and several metrics specific to aSAH are not available despite being desirable. Furthermore, information on comorbidities is restricted to data available in the national inpatient register. To mitigate this, we have used presence of specific prescriptions to expand identification of key comorbidities (e.g. diabetes).

Data quality is another important aspect in all register-based research and can markedly influence results whether incorrect data are analysed. In the current study, we have used domain knowledge in the field of neuro-intensive care and neurosurgery, along with extensive quality assessment of data to guide data handling and variable selection. The degree and distribution of missing data have been investigated (Supplementary Materials) and imputation performed to reduce the risk of this affecting our results. Additionally, avoidance of variables with known poor data quality has been made possible by consulting with experts with experience in constructing and using the register sources. By maintaining a stringent methodology throughout, we are convinced that these issues have been adequately addressed.

Finally, each analysis carries its own set of limitations. In the current study we have used propensity score matching, a method always limited by the quality of matching. Therefore, we assessed several matching specifications prior to analysis and investigated the resulting covariate balance using multiple metrics (Supplementary Materials). Additionally, we attempted to improve the estimation of propensity scores by using an established machine learning classification method of high quality. While the resulting balance will never achieve the level of a fully randomised cohort, we are confident that our efforts have resulted in a matching of sufficient quality to enable interpretation of the results.

## Conclusions

In this study, we demonstrate a significantly increased risk of antidepressant treatment, and most likely also mood disorders, 1 year after ICU admission for aSAH. Importantly, we also show that this increased risk is approximately 33% higher than what would be expected as a consequence of intensive care alone, supporting an inherent pathophysiologic effect of aSAH on the development on mood disorders. Additionally, we are able to translate this increased risk into a statistically significant increase in sick leave, highlighting the socio-economic impact of the disease.

## Supplementary Information

Below is the link to the electronic supplementary material.

Supplementary file1 (HTML 5435 KB)

## Data Availability

Raw data are available from the authors upon reasonable request and following consideration of appropriate legislative procedures regarding confidentiality.

## Abbreviations

**aSAH**: Aneurysmal subarachnoid haemorrhage
**AD**: Antidepressant
**ATT**: Average treatment effect in the treated
**AUROC**: Area under the receiver operating characteristic curve
**ASMD**: Absolute standardised mean difference
**CCI**: Charlson Comorbidity Index
**CDR**: Cause of Death Register
**DP**: Disability pension
**eCDF**: Estimated cumulative distribution function
**GCS**: Glasgow Coma Scale
**GOS**: Glasgow Outcome Scale
**ICU**: Intensive care unit
**ICU LOS**: ICU length of stay
**LISA**: Longitudinal Integrated Database for Health Insurance and Labour Market Studies
**MAR**: Missing at random
**MCAR**: Missing completely at random
**MiDAS**: Microdata for Analysis of Social Insurance
**ML**: Machine learning
**MICE**: Multiple imputation using chained equations
**mRS**: Modified Rankin Scale
**NPR**: National Patient Register
**PDR**: Prescribed Drug Register
**PSM**: Propensity score matching
**RF**: Random Forest
**ROC**: Receiver operating characteristic
**SA**: Sickness absence
**SAP****S ****3**: Simplified Acute Physiology Score 3
**SIR**: Swedish Intensive Care Register
**SQL**: Structured query language
**STROBE**: Strengthening the Reporting of Observational Studies in Epidemiology

## Code Availability

The code is available from the public repository at github.com/ryanfalckjones/epinic-antidepressants-public.

## Clinical Trial Registration

NCT07052656, prospectively registered.
