# Supplementary material for: Increased Antidepressant Use after Intensive Care for Aneurysmal Subarachnoid Haemorrhage: A Machine-Learning-Enhanced Propensity-Matched Nationwide Cohort Study
Source: Neurocrit Care. 2026 May 4;45(2):673–86. doi: 10.1007/s12028-026-02517-z (PMC13642405; doi:10.1007/s12028-026-02517-z)
Supplement: Supplementary file 1 [file 12028_2026_2517_MOESM1_ESM.html]

Supplementary Materials


# Supplementary Materials

### 1. Description of population registers

| Data Source | Description |
| --- | --- |
| Personal identification number (1) | Linking of all data sources was performed using the Swedish personal identification number. This number is assigned to all inhabitants in Sweden at birth or immigration and is an essential part of Swedish records and typically all records are identified using this identification number. |
| Intensive Care Register (2) | The Swedish Intensive Care Register, in Swedish “Svenska Intensivvårdsregistret”, is a quality-assurance register maintained by the organisation with the same name. It consists of data reported from most ICU-wards in Sweden but is subject to reporting practices at each institution. Contains baseline demographics and time-stamps for admission and discharge, along with physiologic scores on admission, specific treatments given in the ICU and certain complications. |
| National Patient Register (4) | The National Patient Register (NPR), in Swedish “Patientregistret”, is maintained by the Swedish National Board of Health and Welfare (Socialstyrelsen) and consists of all documented hospital admissions in Sweden, alongside outpatient appointments to secondary and tertiary care facilities. The NPR also contains all documented interventions performed during the corresponding admission, identified using a KVÅ-code (3). Currently, primary care is not included in the NPR. Data used in the study consists of documented ICD diagnoses (both primary and secondary diagnoses associated with the admission) along with dates of admission and discharge from hospital or appointment date. Data from the NPR was extracted for a period of 5 years prior and 2 years following the date of index admission for each patient. |
| National Cause of Death Register (5) | The National Cause of Death Register (in Swedish “Dödsorsaksregistret”) is maintained by the Swedish National Board of Health and Welfare (Socialstyrelsen) and consists of time and date for all documented deaths in Sweden, along with the ICD-diagnoses responsible for the death in the order specified by the physician pronouncing death. Data from the register was used to validate date of death for patients deceased during the study period. |
| Prescribed Drug Register (6) | The Prescribed Drug Register (in Swedish “Läkemedelsregistret”) is maintained by the Swedish National Board of Health and Welfare (Socialstyrelsen) and consists of the date of prescription and dispensation of all prescribed medications in Sweden, along with the respective Anatomical Therapeutic Chemical (ATC) classification (7). It does not contain information on over-the-counter medications as these are not documented in any way. In the present study, data from the Prescribed Drug Register was used to validate certain diagnoses and to increase identification of pre-existing comorbidities not documented in the NPR, i.e. cases only treated in primary care facilities. |
| Longitudinal Integrated Database for Health Insurance and Labour Market Studies (LISA) (8) | LISA is a population register maintained by Statistics Sweden, and contains data on education level and employment of all inhabitants. Data is stratified per calendar year. |
| MiDAS (9) | MiDAS (MikroData för Analys av Socialförsäkringen) is a register maintained by the Swedish Social Insurance Agency and contains data on sick absence, disability pensions, and parental leave for all inhabitants. Data in this register is available by specific dates and can thus be used for analyses of various types of leave in relation to index dates. |
| References | 1. Lunde AS, Lundeborg S, Lettenstrom GS, Thygesen L, Huebner J. The person-number systems of Sweden, Norway, Denmark, and Israel. Vital Health Stat 2. 1980(84):1-59. 2. Svenska Intensivvårdsregistret. [Register]. Karlstad, Sweden: Svenska Intensivvårdsregistret; [Available from: https://www.icuregswe.org/] 3. National Board of Health and Welfare. Klassifikation av vårdåtgärder (KVÅ) [Website]. [Available from: https://www.socialstyrelsen.se/statistik-och-data/klassifikationer-och-koder/kva/] 4. National Board of Health and Welfare. National Patient Register [Register]. [Available from: https://www.socialstyrelsen.se/en/statistics-and-data/registers/national-patient-register/] 5. National Board of Health and Welfare. Dödsorsaksregistret  [Available from: https://www.socialstyrelsen.se/statistik-och-data/register/dodsorsaksregistret/] 6. National Board of Health and Welfare. National Prescribed Drug Register [Register]. [Available from: https://www.socialstyrelsen.se/en/statistics-and-data/registers/national-prescribed-drug-register/] 7. World Health Organization. Anatomical Therapeutic Chemical (ATC) Classification [Website]. [Available from: https://www.who.int/tools/atc-ddd-toolkit/atc-classification] 8. Statistics Sweden. Longitudinal Integrated Database for Health Insurance and Labour Market Studies (LISA) [Register]. [Available from: https://rut.registerforskning.se/metadatakatalog/register/9abecb9a-a3c3-49ba-8dd4-8e45b5b1839f/] 9. Swedish Social Insurance Agency. MiDAS [Register]. [Available from: https://www.forsakringskassan.se/statistik-och-analys/bestallning-av-individdata-och-statistik/bestallning-av-individdata-for-forskning] |

### 2. ATC-codes used for expansion of comorbidities

| ATC-code | Signals the following comorbidity |
| --- | --- |
| A10 | Diabetes |
| C08C | Hypertension |
| C03A | Hypertension |
| C10 | Hyperlipidaemia |
| B05D | Chronic Kidney Disease |
| B05Z | Chronic Kidney Disease |
| N06D | Dementia |
| C10D | Ischaemic Heart Disease |
| C01A | Congestive Heart Failure |
| N06BA | ADHD |

### 3. Source code-snippet for creation of CTE identifying aSAH patients

```
asah AS (
    SELECT
        P.HADM_ID
    FROM
        PAR_HADM P
    LEFT JOIN
        DORS D ON P.LopNr = D.LopNr
    WHERE (
            (
              (
                P.Diagnos LIKE "I60%" -- Note the placement of the wildcard
                OR 
                (
                  (P.Op LIKE "%AAC00%" OR P.Op LIKE "%AAL00%") 
                  AND P.Diagnos NOT LIKE "I671%"
                )
              )
              AND P.Diagnos NOT LIKE "%S06%"
            )
            OR 
            (
              P.Diagnos LIKE "I60%" 
              AND P.Diagnos LIKE "%S06%" 
              AND (P.Op LIKE "%AAC00%" OR P.Op LIKE "%AAL00%")
            )
            OR
            (
              D.Ulorsak LIKE "I60%"
              AND 
              JULIANDAY(strftime('%Y-%m-%d', 
                                 substr(D.DODSDAT, 1, 4) || '-' || 
                                 substr(D.DODSDAT, 5, 2) || '-' || 
                                 substr(D.DODSDAT, 7, 2) || 
                                 ' 00:00:00')) - JULIANDAY(date(P.INDATUM * 86400,
                                                        'unixepoch')) <= 30
              AND P.Diagnos NOT LIKE "%S06%"
              AND P.Diagnos NOT LIKE "%Q28%"
            )
        )
)
```

### 4. Distribution of demographic variables in study cohort

**Figure 4.1** Distribution of demographic variables in study cohort. A-D: Distribution of categorical variables in pt’s with and without AD-treatment at 1 yr. E: Density plot and QQ-plot of age in the two outcome groups demonstrating roughly normal distribution. F: Density plot of ICU LOS demonstrating extreme right-skew (left), stacked chart of distribution of categories after stratification into four similarly sized groups (right). G: Density plot and QQ-plot of SAPS 3 score demonstrating roughly normal distribution.

### 5. Missing Data

Initial examination of data revealed a low degree of missing data. Only three variables contained missing data: education level, initial consciousness level, and SAPS score. The degree of missingness for each calendar year was examined in Supplementary Table 2 and overall, missing data was evenly distributed over time. Data regarding education level was completely missing for the latter half of 2022 and all of 2023 due to this data not being extracted. There was also a slight increase in missingness for the other two variables in the final years of the study.

| N (%) of missing observations by calendar year | | | | | | | | | | | |
| --- | --- | --- | --- | --- | --- | --- | --- | --- | --- | --- | --- |
| **Characteristic** | **2013**  N = 327^1^ | **2014**  N = 328^1^ | **2015**  N = 293^1^ | **2016**  N = 332^1^ | **2017**  N = 358^1^ | **2018**  N = 292^1^ | **2019**  N = 328^1^ | **2020**  N = 318^1^ | **2021**  N = 275^1^ | **2022**  N = 298^1^ | **2023**  N = 270^1^ |
| Education Level | 5 (1.5%) | 1 (0.3%) | 4 (1.4%) | 2 (0.6%) | 9 (2.5%) | 7 (2.4%) | 7 (2.1%) | 4 (1.3%) | 4 (1.5%) | 136 (46%) | 270 (100%) |
| Initial Consciousness Level | 5 (1.5%) | 4 (1.2%) | 1 (0.3%) | 14 (4.2%) | 8 (2.2%) | 6 (2.1%) | 12 (3.7%) | 3 (0.9%) | 15 (5.5%) | 29 (9.7%) | 48 (18%) |
| SAPS 3-Score | 0 (0%) | 0 (0%) | 0 (0%) | 12 (3.6%) | 0 (0%) | 1 (0.3%) | 1 (0.3%) | 0 (0%) | 14 (5.1%) | 24 (8.1%) | 36 (13%) |
|  |  |  |  |  |  |  |  |  |  |  |  |
| --- | --- | --- | --- | --- | --- | --- | --- | --- | --- | --- | --- |
| ^1^ n (%) | | | | | | | | | | | |

**Table 5.1** Proportion of missing data over time.

Examination of regional differences in degree of missing data across initial admitting ICU revealed certain centres with a high proportion of missing variables, with one centre not reporting SAPS or Consciousness Level.

##### Education

**Figure 5.1** Proportion of missing education data per initial ICU for variables with >0 missing.

##### SAPS 3 Scores

**Figure 5.2** Proportion of missing SAPS 3 scores per initial ICU for variables with >0 missing

Certain centres with a high proportion of missing data are, in fact, very small ICU’s and therefore the missing data is not expected to significantly influence the estimates. Examining centres with five or more missing entries revealed five ICU’s of concern.

**Figure 5.3** Missing data per initial ICU for variables with at least 5 missing entries.

The missing data from these centres was subsequently split by calendar year. This analysis showed Uppsala NIVA highly contributing to missing data in the final three years, in line with the increase in missingness observed.

**Figure 5.4** Missing data per initial ICU for variables with at least 5 missing entries per calendar year.

The figures above revealed that Uppsala NIVA only reported to the ICU-register during the final three years of the study and always with missing SAPS-scores. Additionally, in 2023 Lund NIVA only reported missing SAPS-scores. By excluding Uppsala NIVA and Lund NIVA, the latter only for the calendar year 2023, the degree of missing data regarding SAPS becomes evenly distributed across the entire period 2012-2023.

| N (%) of missing observations by calendar year | | | | | | | | | | | |
| --- | --- | --- | --- | --- | --- | --- | --- | --- | --- | --- | --- |
| **Characteristic** | **2013**  N = 327^1^ | **2014**  N = 328^1^ | **2015**  N = 293^1^ | **2016**  N = 332^1^ | **2017**  N = 358^1^ | **2018**  N = 292^1^ | **2019**  N = 328^1^ | **2020**  N = 318^1^ | **2021**  N = 261^1^ | **2022**  N = 274^1^ | **2023**  N = 230^1^ |
| Initial Consciousness Level | 5 (1.5%) | 4 (1.2%) | 1 (0.3%) | 14 (4.2%) | 8 (2.2%) | 6 (2.1%) | 12 (3.7%) | 3 (0.9%) | 1 (0.4%) | 5 (1.8%) | 12 (5.2%) |
| SAPS 3-Score | 0 (0%) | 0 (0%) | 0 (0%) | 12 (3.6%) | 0 (0%) | 1 (0.3%) | 1 (0.3%) | 0 (0%) | 0 (0%) | 0 (0%) | 0 (0%) |
|  |  |  |  |  |  |  |  |  |  |  |  |
| --- | --- | --- | --- | --- | --- | --- | --- | --- | --- | --- | --- |
| ^1^ n (%) | | | | | | | | | | | |

**Table 5.2** Missing data per calendar year after excluding Uppsala NIVA and Lund NIVA, the latter only for 2023.

Examining the distribution of demographic variables along with the incidence of the primary outcome between Uppsala NIVA and Lund NIVA, the latter only for 2023, versus the remeaning cohort revealed a borderline significant difference in the cumulative incidence of AD-treatment at 1 year. Furthermore, reported predictors such as prior hypertension and invasive ventilation during ICU-admission were significantly different.

##### Distribution of primary outcome and demographic variables between centres with high missingness versus other centres

| **Characteristic** | **NIVA Uppsala + Lund**  N = 78^1^ | **Other**  N = 3,341^1^ | **p-value**^2^ |
| --- | --- | --- | --- |
| ad\_treatment | 13 (17%) | 859 (26%) | 0.070 |
| Female | 46 (59%) | 1,940 (58%) | 0.9 |
| Age | 55 (15) | 58 (13) | 0.053 |
| edu |  |  | 0.011 |
| Primary & Lower Secondary | 3 (11%) | 621 (21%) |  |
| Upper Secondary | 10 (36%) | 1,414 (48%) |  |
| Tertiary | 13 (46%) | 879 (30%) |  |
| Research Degree | 2 (7.1%) | 28 (1.0%) |  |
| Unknown | 50 | 399 |  |
| Hypertension | 25 (32%) | 733 (22%) | 0.034 |
| Diabetes | 5 (6.4%) | 200 (6.0%) | 0.8 |
| Mood Disorder | 0 (0%) | 26 (0.8%) | >0.9 |
| Anxiety Disorder | 0 (0%) | 16 (0.5%) | >0.9 |
| Psychotic Disorder | 0 (0%) | 5 (0.1%) | >0.9 |
| Substance Abuse | 1 (1.3%) | 57 (1.7%) | >0.9 |
| Charlson Comorbidity Index |  |  | 0.2 |
| 0 | 59 (76%) | 2,727 (82%) |  |
| 1 | 11 (14%) | 411 (12%) |  |
| >1 | 8 (10%) | 203 (6.1%) |  |
| SAPS\_consciousness\_level |  |  | >0.9 |
| I (GCS ≥13) | 3 (75%) | 2,291 (70%) |  |
| II (GCS 7-12) | 1 (25%) | 581 (18%) |  |
| III (GCS 6) | 0 (0%) | 112 (3.4%) |  |
| IV (GCS 5) | 0 (0%) | 74 (2.3%) |  |
| V (GCS ≤4) | 0 (0%) | 212 (6.5%) |  |
| Unknown | 74 | 71 |  |
| SAPS\_total\_score | 40 (38, 48) | 48 (44, 57) | 0.2 |
| Unknown | 74 | 14 |  |
| ICU Length of Stay (days) | 6 (7) | 7 (8) | 0.6 |
| Invasive Ventilation during ICU | 32 (41%) | 2,844 (85%) | <0.001 |
|  |  |  |  |
| --- | --- | --- | --- |
| ^1^ n (%); Mean (SD); Median (Q1, Q3) | | | |
| ^2^ Pearson’s Chi-squared test; Wilcoxon rank sum test; Fisher’s exact test | | | |

**Table 5.3** Distribution of primary outcome and demographic variables between centres with high missingness versus other centres.

##### Multiple imputation using chained equations

As a consequence of this difference, a multiple imputation strategy using chained equations was employed on the entire dataset for the logistic regression of risk factors for AD-treatment, with ten iterations being done over five imputations and pooling of analysis in accordance to Rubin’s rules. Convergence of the imputation model was assessed and occurred well before ten iterations had passed.

**Figure 5.5** Examination of multiple imputation model convergance.

### 6. Logistic Regression

The calculation of the SAPS 3 score includes weights from patient age and consciousness level on admission. To avoid entering these variables into the logistic regression model twice, an adjusted SAPS 3 score was created by dropping the weights from these two variables using the following calculation:

\[
\text{Adjusted SAPS} = \text{SAPS} - (P\_{\text{age}} + P\_{\text{GCS}})
\]

where

\[
P\_{\text{age}} =
\begin{cases}
0 & \text{if age} < 40 \\
5 & \text{if } 40 \leq \text{age} \leq 59 \\
9 & \text{if } 60 \leq \text{age} \leq 69 \\
13 & \text{if } 70 \leq \text{age} \leq 74 \\
15 & \text{if } 75 \leq \text{age} \leq 79 \\
18 & \text{if age} \geq 80
\end{cases}
\]

and

\[
P\_{\text{GCS}} =
\begin{cases}
0 & \text{if GCS} \geq 13 \\
2 & \text{if } 7 \leq \text{GCS} \leq 12 \\
7 & \text{if GCS} = 6 \\
10 & \text{if GCS} = 5 \\
15 & \text{if GCS} \leq 4
\end{cases}
\] \*Note: GCS = Glasgow Coma Scale

Examination of collinearity across potential predictors in the logistic regression was initially done by examining the correlation coefficient between continuous variables, Cramérs V between categorical variables, and the point-biserial correlation coefficient between continuous and categorical variables. These analyses revealed little correlation between predictors. Analyses were inspected for each imputation separately and were almost identical, therefore only the first imputation is reported below.

**Figure 6.1** Correlation matrices for continuous and dichotomous predictors used in logistic regression. Note: Only the matrices for one imputation shown.

| Point-biserial Correlation Coefficients | | | |
| --- | --- | --- | --- |
| variable | age | saps\_adj | yr |
| sex\_female | 0.08589901 | 0.04596403 | -0.013624645 |
| edu | -0.09274870 | -0.03804612 | 0.067052602 |
| Hypertension | 0.25628149 | 0.08530579 | -0.018145698 |
| Diabetes | 0.12670282 | 0.01283204 | 0.022908804 |
| CCIw | 0.16482551 | 0.14500610 | -0.072774456 |
| SAPS\_consciousness\_level | 0.02589926 | 0.40568957 | -0.002742944 |
| CONT\_ICU\_LOS\_DAYS | 0.01462656 | 0.29086742 | 0.067874439 |
| any\_imv | 0.02305248 | 0.24503064 | 0.010234405 |
| psych | -0.01500716 | 0.04246610 | -0.005266800 |

**Table 6.1** Correlation matrix for point-biserial correlation coefficients. Note: Only the matrices for one imputation shown.

An initial univariate logistic regression was subsequently fitted to the data for the purpose of examining potential covariates.

| Univariate Logistic Regression | | | | | | | | | | |
| --- | --- | --- | --- | --- | --- | --- | --- | --- | --- | --- |
| **Characteristic** | Imputation 1 | | Imputation 2 | | Imputation 3 | | Imputation 4 | | Imputation 5 | |
|  | **OR** | **95% CI** | **OR** | **95% CI** | **OR** | **95% CI** | **OR** | **95% CI** | **OR** | **95% CI** |
| Female Sex | 1.27 | 1.09, 1.49 | 1.27 | 1.09, 1.49 | 1.27 | 1.09, 1.49 | 1.27 | 1.09, 1.49 | 1.27 | 1.09, 1.49 |
| Age | 1.00 | 0.99, 1.00 | 1.00 | 0.99, 1.00 | 1.00 | 0.99, 1.00 | 1.00 | 0.99, 1.00 | 1.00 | 0.99, 1.00 |
| Education Level |  |  |  |  |  |  |  |  |  |  |
| Primary & Lower Secondary | — | — | — | — | — | — | — | — | — | — |
| Upper Secondary | 1.07 | 0.88, 1.31 | 1.09 | 0.90, 1.34 | 1.08 | 0.88, 1.32 | 1.12 | 0.91, 1.37 | 1.07 | 0.88, 1.31 |
| Tertiary | 0.86 | 0.69, 1.08 | 0.93 | 0.74, 1.16 | 0.91 | 0.73, 1.14 | 0.98 | 0.78, 1.22 | 0.94 | 0.75, 1.18 |
| Research Degree | 0.49 | 0.18, 1.11 | 0.46 | 0.16, 1.11 | 0.47 | 0.16, 1.13 | 0.53 | 0.18, 1.27 | 0.46 | 0.16, 1.10 |
| Hypertension | 1.07 | 0.89, 1.28 | 1.07 | 0.89, 1.28 | 1.07 | 0.89, 1.28 | 1.07 | 0.89, 1.28 | 1.07 | 0.89, 1.28 |
| Diabetes | 1.10 | 0.80, 1.51 | 1.10 | 0.80, 1.51 | 1.10 | 0.80, 1.51 | 1.10 | 0.80, 1.51 | 1.10 | 0.80, 1.51 |
| Charlson Comorbidity Index |  |  |  |  |  |  |  |  |  |  |
| 0 | — | — | — | — | — | — | — | — | — | — |
| >1 | 1.14 | 0.82, 1.56 | 1.14 | 0.82, 1.56 | 1.14 | 0.82, 1.56 | 1.14 | 0.82, 1.56 | 1.14 | 0.82, 1.56 |
| 1 | 1.65 | 1.32, 2.05 | 1.65 | 1.32, 2.05 | 1.65 | 1.32, 2.05 | 1.65 | 1.32, 2.05 | 1.65 | 1.32, 2.05 |
| Consciousness Level |  |  |  |  |  |  |  |  |  |  |
| I (GCS ≥13) | — | — | — | — | — | — | — | — | — | — |
| II (GCS 7-12) | 2.22 | 1.83, 2.69 | 2.20 | 1.81, 2.66 | 2.26 | 1.87, 2.75 | 2.27 | 1.87, 2.76 | 2.24 | 1.85, 2.72 |
| III (GCS 6) | 2.42 | 1.63, 3.56 | 2.42 | 1.64, 3.54 | 2.49 | 1.68, 3.67 | 2.58 | 1.75, 3.78 | 2.53 | 1.71, 3.72 |
| IV (GCS 5) | 2.60 | 1.62, 4.12 | 2.67 | 1.66, 4.24 | 2.91 | 1.81, 4.64 | 2.94 | 1.84, 4.66 | 2.70 | 1.69, 4.27 |
| V (GCS ≤4) | 2.29 | 1.71, 3.04 | 2.49 | 1.86, 3.33 | 2.44 | 1.82, 3.24 | 2.36 | 1.76, 3.14 | 2.31 | 1.72, 3.08 |
| Year | 0.96 | 0.94, 0.99 | 0.96 | 0.94, 0.99 | 0.96 | 0.94, 0.99 | 0.96 | 0.94, 0.99 | 0.96 | 0.94, 0.99 |
| ICU LOS (days) |  |  |  |  |  |  |  |  |  |  |
| [0,1] | — | — | — | — | — | — | — | — | — | — |
| (1,4] | 0.95 | 0.75, 1.21 | 0.95 | 0.75, 1.21 | 0.95 | 0.75, 1.21 | 0.95 | 0.75, 1.21 | 0.95 | 0.75, 1.21 |
| (4,11] | 1.01 | 0.80, 1.26 | 1.01 | 0.80, 1.26 | 1.01 | 0.80, 1.26 | 1.01 | 0.80, 1.26 | 1.01 | 0.80, 1.26 |
| (11,80.1] | 2.10 | 1.70, 2.60 | 2.10 | 1.70, 2.60 | 2.10 | 1.70, 2.60 | 2.10 | 1.70, 2.60 | 2.10 | 1.70, 2.60 |
| Invasive Mechanical Ventilation | 1.60 | 1.28, 2.03 | 1.60 | 1.28, 2.03 | 1.60 | 1.28, 2.03 | 1.60 | 1.28, 2.03 | 1.60 | 1.28, 2.03 |
| Prior Psychiatric Condition | 1.74 | 1.12, 2.66 | 1.74 | 1.12, 2.66 | 1.74 | 1.12, 2.66 | 1.74 | 1.12, 2.66 | 1.74 | 1.12, 2.66 |
| Age-adjusted SAPS 3 score | 1.04 | 1.03, 1.05 | 1.04 | 1.03, 1.05 | 1.04 | 1.03, 1.05 | 1.04 | 1.03, 1.05 | 1.04 | 1.03, 1.05 |
|  |  |  |  |  |  |  |  |  |  |  |
| --- | --- | --- | --- | --- | --- | --- | --- | --- | --- | --- |
| Abbreviations: CI = Confidence Interval, OR = Odds Ratio | | | | | | | | | | |

**Table 6.2** Univariate logistic regression model across each imputation evaluating the OR and 95% CI for each covariate on the cumulative risk of antidepressant treatment at one year after ICU admission.

#### Model diagnostics for multivariable logistic regression model

To assess multicolliniarity in the final multivariable model, a multivariable model was fitted and generalised variance inflation factors (GVIF) assessed. The model demonstrated negligible GVIF for all predictors and used for analysis.

| Variance Inflation Factors for Logistic Regression | | | | | |
| --- | --- | --- | --- | --- | --- |
| Predictor | GVIF\_imp\_1 | GVIF\_imp\_2 | GVIF\_imp\_3 | GVIF\_imp\_4 | GVIF\_imp\_5 |
| sex\_female | 1.026655 | 1.027141 | 1.025973 | 1.026294 | 1.026805 |
| age | 1.118368 | 1.118144 | 1.119440 | 1.116367 | 1.119263 |
| edu | 1.036738 | 1.035780 | 1.032057 | 1.034459 | 1.041577 |
| Hypertension | 1.235033 | 1.238108 | 1.235775 | 1.235450 | 1.236221 |
| Diabetes | 1.074993 | 1.076411 | 1.075158 | 1.075971 | 1.076192 |
| CCIw | 1.268425 | 1.270016 | 1.270960 | 1.270616 | 1.268917 |
| SAPS\_consciousness\_level | 1.450408 | 1.450435 | 1.463143 | 1.462749 | 1.458472 |
| CONT\_ICU\_LOS\_DAYS | 1.324832 | 1.314041 | 1.320519 | 1.322334 | 1.322209 |
| any\_imv | 1.112785 | 1.111096 | 1.111807 | 1.115453 | 1.110856 |
| psych | 1.037931 | 1.036136 | 1.035348 | 1.036718 | 1.036547 |
| saps\_adj | 1.393678 | 1.413146 | 1.404720 | 1.409882 | 1.395029 |
| yr | 1.028860 | 1.028456 | 1.028628 | 1.031243 | 1.029994 |

**Table 6.3** Generalised Variance Inflation Factors for each predictor in the multivariable logistic regression model across all imputations.

Subsequently, we proceeded to perform diagnostics on the logistic regression model. Initially, the assumption of a linear association between the log odds and the predictor value was examined and found to be relatively well met.

**Figure 6.2** Association between log odds and the predictor value for continuous variables.

Following this, two binned residual plots were created, one for expected values vs avg. residuals and the second comparing age and the avg. residual. The plots were inspected for all imputations and found to be similar, therefore only one plot is reported. Influential values were investigated by plotting Cook’s distance and subsequently the std. residual vs. leverage.

**Figure 6.3** Top-left: Binned residual plot for multivariable model on 1st imputation comparing expected values and the avg. residuals. Top-right: Binned residual plot for multivariable model on 1st imputation comparing age and the avg. residuals. Bottom-left: Cook’s distance plot for multivariable model on 1st imputation with points marked representing the 10 highest Cook’s distance values. Bottom-right: Plot for multivariable model on 1st imputation comparing leverage against the std. Pearson residuals with points marked representing the 10 highest Cook’s distance values.

### 7. Sensitivity Analyses

#### Effect of varying time required to be considered AD-naïve

To investigate any potential bias occurring due to the definition of the study cohort, a sensitivity analysis was performed by increasing the length of the required AD-free period before ICU admission from 6 monts to 9 months and 1 year. The result was plotted as the cumulative incidence of AD-treatment at 1 year (the primary outcome) along with 95% CI’s using Wald’s method. There was no significant difference in the primary outcome regardless of the length of the AD-free period, as demonstrated by a Chi-Squared test.

**Figure 7.1** Effect of changing the required period before ICU admission with no AD-prescription on the cumulative incidence of AD-prescriptions within 1 year after aSAH.

#### Negative pharmacologic controls to assess follow-up bias

Since follow-up for patients with aSAH tends to be very detailed, there is a clear risk that the increase in AD-prescription we identify after aSAH is simply a result of more healthcare attention after the event. To investigate this, we extracted prescriptions of two other drugs not assumed to be related to aSAH and plotted over time before and after ICU-admission for aSAH. This analysis showed a linear association between time and treatment for these drugs, clearly differing from the stark increase in AD-prescriptions after aSAH. The absence of a sudden increase in other prescriptions supports the hypothesis that the increase in AD-prescriptions after aSAH is not only due to improved follow-up of these patients.

**Figure 7.2** Point prevalence graph of three different prescriptions relative to ICU-admission. By querying the Prescribed Drugs Register each day between -2 years to +2 years of ICU admission for aSAH, a point prevalence of having an active prescription of a substance was calculated. Active prescription was defined as having received a prescription within 90 days prior. Smoothing curves are overlayed for each substance with antidepressants using a Generalised Additive Model and the two other substances using a Linear Model.

### 8. Performance Metrics for Propensity Score Model

The Random Forest propensity score estimation model was evaluated using 5-fold cross-validation with near identical ROC-curves for each fold (not shown). Final performance metrics including accuracy, Brier class, and AUROC were plotted for each imputation of the study cohort joined with the control cohort following listwise deletion of missing data in this cohort.

**Figure 8.1** Performance metrics for the propensity score estimation model across all imputations of the study cohort joined with the control cohort following listwise deletion.

The hyperparameters of the model were also tuned using a Latin Hypercube method with the goal of maximising the AUROC. The results of tuning are presented as scatterplots across iterations.

**Figure 8.2** Results of hyperparameter tuning across iterations

### 9. Matching Diagnostics

#### Balance diagnostics

Various matching specifications were assessed for covariate balance using both the Absolute Standardized Mean Difference and the comparison of distributions using the Kolmogorov-Smirnov (KS) statistic, both described below.

The absolute standardized mean difference (ASMD) is defined as: \[
ASMD = \dfrac{|\bar{x}\_T - \bar{x}\_C|}{\sqrt{\dfrac{s\_T^2 + s\_C^2}{2}}}
\]

Where \(\bar{x}\_T\) and \(\bar{x}\_C\) are the means in the treatment and control groups, and \(s\_T^2\), \(s\_C^2\) are their variances.

The KS-statistic is defined as:

For two empirical CDFs, \(F\_1(x)\) and \(F\_2(x)\), the KS statistic is

\[
D = \sup\_x \big| F\_1(x) - F\_2(x) \big|
\]

Where:

- \(F\_1(x)\) = CDF of the treatment group
- \(F\_2(x)\) = CDF of the control group
- \(\sup\_x\) = supremum (maximum) over all values of \(x\)

The balance plots for three different matching ratios are presented below.

**Figure 9.1** Covariate balance diagnostics before and after Nearest-Neighbour matching demonstrating a notable reduction of the Absolute Standardized Mean Difference and Kolmogorov-Smirnov Statistic. A = 3:1-matching, B = 4:1-matching, C = 5:1-matching. Dashed vertical line at ASMD = 0.1. Ordinal covariates are entered into the model as orthogonal polynomial splines in order to retain ordinal non-linear effects. For underlying groups, see Table 9.1.

Additionally, further balance diagnostics were performed between matching specifications before deciding upon 3:1 matching. Initially, the estimated Cumulative Distribution Functions were visually reviewed for continuous covariates.

**Figure 9.2** Estimated Cumulative Distribution Functions for continuous covariates before and after matching using various matching ratios.

Subsequently, the balance statistics for the matching ratios for all covariates and their squares were summarised.

**Figure 9.3** Comparison of balance statistics across matching ratios.

Based on the above figures, the optimal matching ratio was judged to be 3:1 Nearest-Neighbour Matching and therefore employed.

#### Baseline characteristics in matched cohort

The distribution of baseline characteristics were also assessed before and after 3:1 matching showing a substantial difference prior to matching which was substantially reduced after matching.

| **Characteristic** | **Matched Controls**  N = 10,257^1^ | **Study Cohort**  N = 3,419^1^ | **All Controls**  N = 95,816^1^ |
| --- | --- | --- | --- |
| Female | 5,958 (58%) | 1,986 (58%) | 37,493 (39%) |
| Age | 58 (15) | 58 (13) | 57 (19) |
| Education Level |  |  |  |
| Primary & Lower Secondary | 1,841 (18%) | 717 (21%) | 33,101 (35%) |
| Upper Secondary | 4,968 (48%) | 1,621 (47%) | 43,538 (45%) |
| Tertiary | 3,341 (33%) | 1,040 (30%) | 18,613 (19%) |
| Research Degree | 107 (1.0%) | 41 (1.2%) | 564 (0.6%) |
| Hypertension | 1,831 (18%) | 758 (22%) | 23,898 (25%) |
| Diabetes | 577 (5.6%) | 205 (6.0%) | 12,194 (13%) |
| Psychiatric Cond. | 368 (3.6%) | 92 (2.7%) | 15,744 (16%) |
| Charlson Comorbidity Index |  |  |  |
| 0 | 8,385 (82%) | 2,786 (81%) | 58,511 (61%) |
| 1 | 1,373 (13%) | 422 (12%) | 14,303 (15%) |
| >1 | 499 (4.9%) | 211 (6.2%) | 23,002 (24%) |
| Initial Consciousness Level |  |  |  |
| I (GCS ≥13) | 7,443 (73%) | 2,408 (70%) | 75,180 (78%) |
| II (GCS 7-12) | 1,748 (17%) | 597 (17%) | 13,168 (14%) |
| III (GCS 6) | 293 (2.9%) | 114 (3.3%) | 2,256 (2.4%) |
| IV (GCS 5) | 234 (2.3%) | 77 (2.3%) | 884 (0.9%) |
| V (GCS ≤4) | 539 (5.3%) | 223 (6.5%) | 4,328 (4.5%) |
| Adj. SAPS 3-score | 42 (10) | 41 (8) | 40 (11) |
| ICU LOS |  |  |  |
| 0-1 days | 2,495 (24%) | 870 (25%) | 46,018 (48%) |
| 1-4 days | 2,004 (20%) | 766 (22%) | 35,221 (37%) |
| 4-11 days | 3,250 (32%) | 920 (27%) | 10,049 (10%) |
| >11 days | 2,508 (24%) | 863 (25%) | 4,528 (4.7%) |
|  |  |  |  |
| --- | --- | --- | --- |
| ^1^ n (%); Mean (SD) | | | |

**Table 9.1** Patient characteristics in the study cohort alongside the matched and unmatched control cohorts. Here, Full OPIC refers to the entire control cohort *following* listwise deletion due to missing data (i.e. the control cohort entering the matching procedure).

#### Conclusion regarding matching procedure

In conclusion, the 3:1 propensity score matching procedure generated a significantly improved balance with mean ASMD below 0.05 indicating excellent balance. Using this specification, we subsequently proceeded to apply the doubly robust model to estimate the ATT.

### 10. References for R packages used in environment

The R-packages used in analysis are referenced below. Source code is publically available at the repository referenced in the main article.

1.

Robinson D, Hayes A, Couch S. Broom: Convert statistical objects into tidy tibbles [Internet]. 2025. Available from: https://broom.tidymodels.org/

2.

Greifer N. Cobalt: Covariate balance tables and plots [Internet]. 2025. Available from: https://ngreifer.github.io/cobalt/

3.

Barrett T, Dowle M, Srinivasan A, Gorecki J, Chirico M, Hocking T, et al. Data.table: Extension of ‘data.frame‘ [Internet]. 2025. Available from: https://r-datatable.com

4.

R Special Interest Group on Databases (R-SIG-DB), Wickham H, Müller K. DBI: R database interface [Internet]. 2024. Available from: https://dbi.r-dbi.org

5.

Kuhn M, Frick H. Dials: Tools for creating tuning parameter values [Internet]. 2025. Available from: https://dials.tidymodels.org

6.

Wickham H, François R, Henry L, Müller K, Vaughan D. Dplyr: A grammar of data manipulation [Internet]. 2023. Available from: https://dplyr.tidyverse.org

7.

Wickham H, Girlich M, Fairbanks M, Dickerson R. Dtplyr: Data table back-end for dplyr [Internet]. 2025. Available from: https://dtplyr.tidyverse.org

8.

Wickham H. Forcats: Tools for working with categorical variables (factors) [Internet]. 2025. Available from: https://forcats.tidyverse.org/

9.

Vaughan D, Dancho M. Furrr: Apply mapping functions in parallel using futures [Internet]. 2022. Available from: https://github.com/DavisVaughan/furrr

10.

Bengtsson H. Future: Unified parallel and distributed processing in r for everyone [Internet]. 2025. Available from: https://future.futureverse.org

11.

Wickham H, Chang W, Henry L, Pedersen TL, Takahashi K, Wilke C, et al. ggplot2: Create elegant data visualisations using the grammar of graphics [Internet]. 2025. Available from: https://ggplot2.tidyverse.org

12.

Iannone R, Cheng J, Schloerke B, Hughes E, Lauer A, Seo J, et al. Gt: Easily create presentation-ready display tables [Internet]. 2025. Available from: https://gt.rstudio.com

13.

Sjoberg DD, Larmarange J, Curry M, de la Rua E, Lavery J, Whiting K, et al. Gtsummary: Presentation-ready data summary and analytic result tables [Internet]. 2025. Available from: https://github.com/ddsjoberg/gtsummary

14.

Bray A, Ismay C, Chasnovski E, Couch S, Baumer B, Cetinkaya-Rundel M. Infer: Tidy statistical inference [Internet]. 2025. Available from: https://github.com/tidymodels/infer

15.

Spinu V, Grolemund G, Wickham H. Lubridate: Make dealing with dates a little easier [Internet]. 2024. Available from: https://lubridate.tidyverse.org

16.

Arel-Bundock V. Marginaleffects: Predictions, comparisons, slopes, marginal means, and hypothesis tests [Internet]. 2025. Available from: https://marginaleffects.com/

17.

Ho D, Imai K, King G, Stuart E, Greifer N. MatchIt: Nonparametric preprocessing for parametric causal inference [Internet]. 2025. Available from: https://kosukeimai.github.io/MatchIt/

18.

Pishgar F, Greifer N. MatchThem: Matching and weighting multiply imputed datasets [Internet]. 2024. Available from: https://github.com/FarhadPishgar/MatchThem

19.

van Buuren S, Groothuis-Oudshoorn K. Mice: Multivariate imputation by chained equations [Internet]. 2025. Available from: https://github.com/amices/mice

20.

Kuhn M. Modeldata: Data sets useful for modeling examples [Internet]. 2025. Available from: https://modeldata.tidymodels.org

21.

Kuhn M, Vaughan D. Parsnip: A common API to modeling and analysis functions [Internet]. 2025. Available from: https://github.com/tidymodels/parsnip

22.

Wickham H, Henry L. Purrr: Functional programming tools [Internet]. 2025. Available from: https://purrr.tidyverse.org/

23.

Wright MN. Ranger: A fast implementation of random forests [Internet]. 2024. Available from: https://imbs-hl.github.io/ranger/

24.

Falck-Jones R. Rcmswe: Register-based CoMorbidities in SWEdish settings [Internet]. 2025. Available from: https://github.com/ryanfalckjones/rcmswe

25.

Wickham H, Hester J, Bryan J. Readr: Read rectangular text data [Internet]. 2024. Available from: https://readr.tidyverse.org

26.

Kuhn M, Wickham H, Hvitfeldt E. Recipes: Preprocessing and feature engineering steps for modeling [Internet]. 2025. Available from: https://github.com/tidymodels/recipes

27.

Frick H, Chow F, Kuhn M, Mahoney M, Silge J, Wickham H. Rsample: General resampling infrastructure [Internet]. 2025. Available from: https://rsample.tidymodels.org

28.

Müller K, Wickham H, James DA, Falcon S. RSQLite: SQLite interface for r [Internet]. 2025. Available from: https://rsqlite.r-dbi.org

29.

Wickham H, Pedersen TL, Seidel D. Scales: Scale functions for visualization [Internet]. 2025. Available from: https://scales.r-lib.org

30.

Wickham H. Stringr: Simple, consistent wrappers for common string operations [Internet]. 2025. Available from: https://stringr.tidyverse.org

31.

Couch S, Frick H, HvitFeldt E, Kuhn M. Tailor: Iterative steps for postprocessing model predictions [Internet]. 2025. Available from: https://github.com/tidymodels/tailor

32.

Müller K, Wickham H. Tibble: Simple data frames [Internet]. 2025. Available from: https://tibble.tidyverse.org/

33.

Kuhn M, Wickham H. Tidymodels: Easily install and load the tidymodels packages [Internet]. 2025. Available from: https://tidymodels.tidymodels.org

34.

Wickham H, Vaughan D, Girlich M. Tidyr: Tidy messy data [Internet]. 2024. Available from: https://tidyr.tidyverse.org

35.

Wickham H. Tidyverse: Easily install and load the tidyverse [Internet]. 2023. Available from: https://tidyverse.tidyverse.org

36.

Kuhn M. Tune: Tidy tuning tools [Internet]. 2025. Available from: https://tune.tidymodels.org/

37.

Vaughan D, Couch S, Frick H. Workflows: Modeling workflows [Internet]. 2025. Available from: https://github.com/tidymodels/workflows

38.

Frick H, Kuhn M, Couch S. Workflowsets: Create a collection of tidymodels workflows [Internet]. 2025. Available from: https://github.com/tidymodels/workflowsets

39.

Kuhn M, Vaughan D, Hvitfeldt E. Yardstick: Tidy characterizations of model performance [Internet]. 2025. Available from: https://github.com/tidymodels/yardstick

40.

Bengtsson H. A unifying framework for parallel and distributed processing in r using futures. The R Journal [Internet]. 2021;13(2):208–27. Available from: https://doi.org/10.32614/RJ-2021-048

41.

Wickham H. ggplot2: Elegant graphics for data analysis [Internet]. Springer-Verlag New York; 2016. Available from: https://ggplot2.tidyverse.org

42.

Sjoberg DD, Whiting K, Curry M, Lavery JA, Larmarange J. Reproducible summary tables with the gtsummary package. The R Journal [Internet]. 2021;13:570–80. Available from: https://doi.org/10.32614/RJ-2021-053

43.

Couch SP, Bray AP, Ismay C, Chasnovski E, Baumer BS, Çetinkaya-Rundel M. infer: An R package for tidyverse-friendly statistical inference. Journal of Open Source Software. 2021;6(65):3661.

44.

Grolemund G, Wickham H. Dates and times made easy with lubridate. Journal of Statistical Software [Internet]. 2011;40(3):1–25. Available from: https://www.jstatsoft.org/v40/i03/

45.

Arel-Bundock V, Greifer N, Heiss A. How to interpret statistical models using marginaleffects for R and Python. Journal of Statistical Software. 2024;111(9):1–32.

46.

Ho DE, Imai K, King G, Stuart EA. MatchIt: Nonparametric preprocessing for parametric causal inference. Journal of Statistical Software. 2011;42(8):1–28.

47.

Pishgar F, Greifer N, Leyrat C, Stuart E. MatchThem:: Matching and weighting after multiple imputation. The R Journal [Internet]. 2021; Available from: https://journal.r-project.org/archive/2021/RJ-2021-073/

48.

van Buuren S, Groothuis-Oudshoorn K. mice: Multivariate imputation by chained equations in r. Journal of Statistical Software. 2011;45(3):1–67.

49.

Wright MN, Ziegler A. ranger: A fast implementation of random forests for high dimensional data in C++ and R. Journal of Statistical Software. 2017;77(1):1–17.

50.

Kuhn M, Wickham H. Tidymodels: A collection of packages for modeling and machine learning using tidyverse principles. [Internet]. 2020. Available from: https://www.tidymodels.org

51.

Wickham H, Averick M, Bryan J, Chang W, McGowan LD, François R, et al. Welcome to the tidyverse. Journal of Open Source Software. 2019;4(43):1686.
